# Unearthing the transition rates between photoreceptor conformers

**DOI:** 10.1186/s12918-016-0368-y

**Published:** 2016-11-25

**Authors:** Robert W. Smith, Britta Helwig, Adrie H. Westphal, Eran Pel, Maximilian Hörner, Hannes M. Beyer, Sophia L. Samodelov, Wilfried Weber, Matias D. Zurbriggen, Jan Willem Borst, Christian Fleck

**Affiliations:** 1 Laboratory of Systems & Synthetic Biology, Wageningen UR, PO Box 8033, Wageningen, 6700EJ The Netherlands; 2 LifeGlimmer GmbH, Markelstrasse 38, Berlin, 12163 Germany; 3 Laboratory of Biochemistry, PO Box 8128, Wageningen, 6700ET The Netherlands; 4 Spemann Graduate School of Biology and Medicine, University of Freiburg, Albertstrasse 19A, Freiburg, 79104 Germany; 5 Faculty of Biology & BioSS, University of Freiburg, Schänzlestrasse 18, Freiburg, 79104 Germany; 6 Institute of Synthetic Biology, Heinrich Heine University, Universitätsstrasse 1, Düsseldorf, 40225 Germany

**Keywords:** Phytochromes, Photoconversion, Optimisation, Optogenetics

## Abstract

**Background:**

Obtaining accurate estimates of biological or enzymatic reaction rates is critical in understanding the design principles of a network and how biological processes can be experimentally manipulated on demand. In many cases experimental limitations mean that some enzymatic rates cannot be measured directly, requiring mathematical algorithms to estimate them. Here, we describe a methodology that calculates rates at which light-regulated proteins switch between conformational states. We focus our analysis on the phytochrome family of photoreceptors found in cyanobacteria, plants and many optogenetic tools. Phytochrome proteins change between active (*P*_*A*_) and inactive (*P*_*I*_) states at rates that are proportional to photoconversion cross-sections and influenced by light quality, light intensity, thermal reactions and dimerisation. This work presents a method that can accurately calculate these photoconversion cross-sections in the presence of multiple non-light regulated reactions.

**Results:**

Our approach to calculating the photoconversion cross-sections comprises three steps: i) calculate the thermal reversion reaction rate(s); ii) develop search spaces from which all possible sets of photoconversion cross-sections exist, and; iii) estimate extinction coefficients that describe our absorption spectra. We confirm that the presented approach yields accurate results through the use of simulated test cases. Our test cases were further expanded to more realistic scenarios where noise, multiple thermal reactions and dimerisation are considered. Finally, we present the photoconversion cross-sections of an *Arabidopsis* phyB N-terminal fragment commonly used in optogenetic tools.

**Conclusions:**

The calculation of photoconversion cross-sections has implications for both photoreceptor and synthetic biologists. Our method allows, for the first time, direct comparisons of photoconversion cross-sections and response speeds of photoreceptors in different cellular environments and synthetic tools. Due to the generality of our procedure, as shown by the application to multiple test cases, the photoconversion cross-sections and quantum yields of any photoreceptor might now, in principle, be obtained.

**Electronic supplementary material:**

The online version of this article (doi:10.1186/s12918-016-0368-y) contains supplementary material, which is available to authorized users.

## Background

The aim of computational biology is to obtain a better quantitative understanding of biological systems and the principles upon which evolution has constrained cellular networks. Building on such knowledge, networks can be designed that yield certain optimal responses in both natural and synthetically constructed organisms. However, despite the wealth of experimental techniques and data available, biological parameters can only be estimated relative to standard values, if they can be calculated at all. This has increasingly led to the development of optimisation techniques that aim to find the most accurate or probable set of parameters that describe a particular set of data (see [1, 2]). Here, we will focus on one instance where this is a particularly pertinent problem - namely the rates at which a light-regulated protein (photoreceptor) is able to switch between different conformational states.

One such family of photoreceptors is the phytochromes. Phytochromes have been found throughout the cyanobacterial and land plant kingdoms [3, 4]. Each of these proteins respond to light through a similar chromophore-based mechanism [5]. Upon illumination of red light, a conformational change in the chromophore leads to the activation of phytochromes (*P*_*A*_ = active phytochrome) to regulate downstream networks [6, 7]. This process can be reversed, leading to increased populations of inactive phytochrome (*P*_*I*_), by illumination with a second light source of a different wavelength or leaving the protein in the dark, where thermal reactions control the conformational change (thermal reversion) [8–12]. The wavelength of light required for phytochrome inactivation changes between phytochrome species, with plant phytochromes requiring far-red light whilst bacterial and algal phytochromes can be inactivated by wavelengths across the visible light spectrum [4, 11]. The transition rates between conformers is related to the photoconversion cross-sections of the photoreceptor and the light conditions that the photoreceptor is exposed to (i.e. the intensity and spectral composition of the light source) [13, 14]. photoconversion cross-sections are, essentially, the ability of a molecule to absorb a photon of a given wavelength resulting in a conformational change [15]. A range of spectroscopic techniques have been used to observe these state changes [9, 11, 16–18]. For phytochromes, absorption spectra show a relatively large spectral overlap implying the presence of mixed populations of *P*_*A*_, *P*_*I*_ and, in some cases, intermediate conformations (such as Lumi-R [19–21]), co-existing within the protein sample. By using absorption spectra, one can not only observe levels of *P*_*A*_ or *P*_*I*_ within a phytochrome sample, but the level of absorbance can be related to sample concentration, molecular weight and extinction coefficient [10, 11, 13, 15]. The extinction coefficient is defined as the ratio between the photoconversion cross-section of a phytochrome species and its quantum yield, where the quantum yield is the ratio of absorbed photons that lead to a molecular change with the total number of photons that are absorbed [15].

Therefore, photoconversion cross-sections can be inferred directly from absorption spectra if some other parameters are known. This is the case often described by Butler’s model [15]. However, to accurately estimate the photoconversion cross-section and quantum yield of one phytochrome conformational state using this model, an experimental configuration is needed whereby 100% of the population is in a single state. For many phytochromes, no experimental condition can achieve such requirements due to overlapping absorption spectra that are indicative of mixed populations [13, 15]. Previous studies of plant phytochromes from *Avena sativa* have estimated the photoconversion cross-sections and quantum yields by assuming specific percentages of *P*_*A*_ within the phytochrome population under different conditions (∼87% after saturating red light illumination, 100% after far-red illumination), allowing Butler’s model to be solved [10, 11]. We shall refer to these estimates as the ‘Mancinelli spectra’ and ‘Mancinelli quantum yields’ following the review by Alberto Mancinelli [13].

Building on Butler’s model, the Verméglio group has proposed an analytical method to determine the photoconversion cross-sections of phytochrome proteins and the ratio of the quantum yields between the *P*_*A*_ and *P*_*I*_ states within a mixed population [22, 23]. By using absorption spectra obtained as a result of three different illumination conditions, one can calculate the photoconversion cross-sections by solving a system of linear equations [23]. Quantum yields need to be calculated using other methods. However, these analytical equations do not take into account any effects of thermal reversion which, under certain conditions, can play a significant role in the reactions governing phytochrome transitions [14, 24]. Another set of methods have also recently been developed that can estimate the number of subpopulations within a proteins reaction cycle [16–18]. As a result these methods have enabled comparisons of quantum yields between a phytochrome subfamily but does not provide any information about photoconversion cross-sections or transition rates [25]. Thus, neither of these approaches provide the same level of information obtained from the ‘Mancinelli spectra’ that allows researchers to calculate, for any wavelength of light, how the phytochrome populations switch between different states.

An example of a phytochrome protein, whose dynamics have been studied both within its natural context and in optogenetic tools, is phytochrome B (phyB) from *Arabidopsis thaliana* [14, 26–28]. Generally, phyB works as follows regardless of host cell. Once synthesised in the cytosol, phyB covalently binds tetrapyrrole chromophores (phytochromobilin (P *Φ*B) in plants, cyanobacterial phycocyanobilin (PCB) in cyanobacteria and synthetic systems) to form a holoprotein [6, 7]. As with other phytochromes, phyB is activated by red light and inactivated by thermal reversion and/or exposure to far-red light. Upon activation, phyB is able to interact with other proteins, translocate into the nucleus and control downstream processes [27, 29]. These downstream processes include transcription [26, 30], protein degradation [31] and protein sequestration, potentially through the formation of nuclear bodies [27, 32, 33]. In plants, the interactions of phyB eventually lead to the suppression of seedling elongation and inhibition of the vegetative to flowering transition [34].

So far in optogenetics, the truncated N-terminal version of phyB (consisting of the first 650 amino acids and referred to as phyB-N from hereon) has been used [9, 26, 27]. Importantly, phyB-N has been shown to be the most stable construct that still exhibits the correct responses and dynamics to light signals compared to the full-length variant [9, 26, 27, 35] whilst thermal relaxation of phyB-N is slower in vitro than full-length phyB in vivo [8, 12, 28]. Futhermore, compared to the activity of full-length phyB *in planta*, which has been shown to act as a dimer, phyB-N in synthetic systems is believed to function as a monomer [36, 37]. Formation of phyB dimers in plants means that the networks regulating downstream activity are different between natural and orthogonal synthetic systems [28, 38, 39]. Through an increasing number of systems biology studies, the networks and kinetic rates describing phyB’s role in controlling plant development have been estimated and verified [14, 28, 40]. Since these studies have dealt with the full-length phyB protein, they have assumed that the ‘Mancinelli spectra’ are the correct photoconversion cross-sections that determine phyB conformational changes. Despite the ‘Mancinelli spectra’ being calculated using full-length plant phyA, this approximation is justified since plant phyA and phyB molecules respond to light in a similar manner. However, as a wider range of phytochromes and their structural variants are being studied for their photophysical properties and dynamics in natural and optogenetic systems, there is a real need to accurately estimate these photoconversion cross-sections and the rates at which phytochromes switch between conformational states and how efficient these processes are. With such knowledge, one could potentially look to compare and optimise the transition rates of photoreceptors for use in optogenetic networks via protein engineering.

Here, we build on the analytical methods proposed by the Verméglio group to include further phytochrome reactions, such as thermal reversion and dimerisation, to obtain upper limits for the quantum yield ratio and search spaces for the photoconversion cross-sections. Using this information, we then propose an optimisation algorithm that reliably estimates the photoconversion cross-sections and quantum yields of any photoreceptor. The generality of our approach is highlighted by simulating different members of the phytochrome family - including a red/green cyanobacteriochrome and a red/far-red plant phytochrome. First, our output is compared to those obtained by Butler’s model, showing that our algorithm can find the correct set of photoconversion cross-sections and quantum yields when Butler’s model fails. Subsequently we show, for the red/far-red photoreceptor reminiscent of full-length phytochromes found in plants, that our algorithm can find the correct photoconversion cross-sections and quantum yields even in the presence of multiple thermal reversion reactions. Furthermore, the results are insensitive to noise. Finally, our method is applied to the phyB-N protein used in optogenetics and provides the photoconversion cross-sections for this protein for the first time in vitro. Given adequate data, we propose that this procedure can be adopted for any photoreceptor protein and will be of use to those wishing to obtain quantitative understanding of light-regulated processes in natural and synthetic biological systems or comparisons of phytochrome family members and structural variants.

## Methods

### Methodology overview

**Fig. 1:**
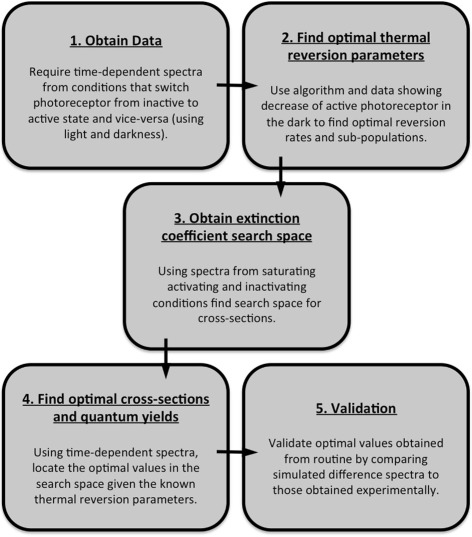
An overview of the method. To obtain photoreceptor photoconversion cross-sections and quantum yields, four computational steps are required once the appropriate data has been obtained (Box 1). First, the parameters for thermal reversion - the rate of reversion and the percentage of the photoreceptor population that experiences this rate - need to be found (Box 2). Given these values, an analytical method is then used to find the search spaces for our optimisation algorithm (Box 3). Once the parameters and search spaces have been obtained, the values can be found using our optimisation algorithm (Box 4). Finally, the optimal values can be verified by comparing simulations to the rest of our available data (Box 5)

Below, the details of our methodology that infers the photoconversion cross-sections and quantum yields of photoreceptor proteins are described. In Fig. 1, an overview of the steps is presented.

In order for the method to work, absorption spectra need to be obtained from different experimental conditions. Importantly, this data needs to illustrate the switching from active to inactive states of the photoreceptor and *vice versa*. For a photoreceptor that responds to a single region of the light spectrum (such as the cryptochromes [41]) spectra need to be obtained from two experiments: under constant darkness after the photoreceptor population has been completely activated by light, and under prolonged periods of activating light after complete inactivation in darkness (see Section 1.4 of Additional file 1). For phytochromes, that respond to two regions of the light spectrum (for example see [11]), data would need to be obtained after initial illumination of red light followed by constant treatment with inactivating light conditions or darkness and *vice versa*, recording the activation of phytochromes in red light after initial exposure to inactivating light.

Ultimately, we aim to simulate absorption spectra, *A*_*s*_, that match experimental data, *A*_*e*_. Simulated absorption spectra are represented by

1 $$\begin{array}{*{20}l} & A_{s} \!({\lambda}|\Omega_{P},\Omega_{E}) \,=\, 2.303{lc}_{tot}\sum_{k=1}^{N}{c^{k}}\!(\boldsymbol{\sigma},\boldsymbol{\Phi},\boldsymbol{\alpha},\boldsymbol{\beta}, \Omega_{P},\Omega_{E})\frac{\sigma_{k}^{\lambda}}{\Phi_{k}}, \\ & \sum_{k=1}^{N}{c^{k}}(\boldsymbol{\sigma},\boldsymbol{\Phi},\boldsymbol{\alpha},\boldsymbol{\beta},\Omega_{P},\Omega_{E}) = n_{c}, \end{array} $$

where *A*_*s*_(*λ*|*Ω*_*P*_,*Ω*_*E*_) is the simulated absorption at wavelength *λ* given a set of tuples describing the preparatory, $\Omega _{P} = \{(t_{P},z_{P}^{\lambda })\}$, and experimental, $\Omega _{E}= \{(t_{E},z_{E}^{\lambda })\}$, light conditions. Here, the light conditions are described by the time of exposure, *t*_*P*_ and *t*_*E*_, in s and the spectral and intensity composition of the light source, $z_{P}^{\lambda }$ and $z_{E}^{\lambda }$, in *μ*mol m ^−2^ s ^−1^. The terms *c* ^*k*^ are time-dependent changes in the fraction of available chromophore, *n*_*c*_, within the photoreceptor monomer or dimer structure that is in state *k*. The parameters $\boldsymbol \sigma = \{\sigma _{1}^{\lambda },\ldots,\sigma _{k}^{\lambda },\ldots,\sigma _{N}^{\lambda }\}$ is the set of wavelength-dependent photoconversion cross-sections (in m^2^/mol) of a sample population switching between states *P*_*I*_ and *P*_*A*_, and the ***Φ***={*Φ*_1_,…,*Φ*_*k*_,…,*Φ*_*N*_} is the set of associated quantum yields of the light-dependent reactions. Furthermore, the *c* ^*k*^’s depend on the parameters related to thermal reversion, where ***α***={*α*_1_,…,*α*_*n*_} is the percentage of the active photoreceptor (*P*_*A*_) population that undergoes a thermal reversion rate of ***β***={*β*_1_,…,*β*_*n*_}, where *n* is the number of *P*_*A*_ species in the population (see below), and the preparatory/experimental light conditions (*Ω*_*P*_, *Ω*_*E*_). In Section 1.2 of Additional file 1, we show the general forms for *c* ^*k*^ used in our study and how this function is altered between different cases.

The total concentration of photoreceptor in our sample, *c*_*tot*_, is defined in mol/L, the term 2.303 is required to relate absorption to measured light fluences and *l* is the light path length through the sample-containing cuvette in cm [15]. For our study, we have a cuvette containing $c_{tot} = \frac {0.34~\text {mg/ml}}{70000~\text {Da}} (\sim \frac {\text {mol}}{\mathrm {L}})$ sample that is 1 cm thick and so, due to light path corrections, we use *l*=1.06 cm as in [15].

The proposed methodology performs three steps:

1. The parameters for thermal reversion kinetics are found, such as the rate of reversion, ***β***, and the percentage of the population, ***α***, that undergoes reversion at this rate (Fig. 1, Box 2). Importantly, these parameters play a role in determining *c* ^*k*^.
2. Appropriate search spaces for the extinction coefficients, $\epsilon _{k}^{\lambda } = \sigma _{k}^{\lambda }/\Phi _{k}$, and the upper bound of the quantum yield ratio, *R* ^max^, are found numerically, given the thermal reversion parameters, ***α*** and ***β*** (Fig. 1, Box 3).
3. photoconversion cross-sections, $\sigma _{k}^{\lambda }$, and quantum yields, *Φ*_*k*_, are obtained by comparing simulations described by Eq. (1) to measured time-dependent absorption spectra (Fig. 1, Box 4).

The resulting photoconversion cross-sections and quantum yield values should then be validated against known data. In our examples, we use either the known values (where simulated data is used) or our available datasets (in cases where biological data is used; Fig. 1, Box 5). This section will present the details of each step of the approach and the elucidation of photoconversion cross-sections and quantum yields. In the results section, we will show the output from a series of test cases before using data obtained for phyB-N.

### Step 1: Determination of thermal reversion rates

In order to determine the thermal reversion rates of a photoreceptor, data needs to be obtained that details the inactivation of the photoreceptor. To do this, the sample must be placed in constant darkness after a saturating period of activating light (see Section 2 in Additional file 1). The percentage decrease of active photoreceptor can then be obtained from

2 $$ {{\begin{aligned} {E_{t}^{D}} =\! \frac{A_{e}\left(\!\lambda\left|\left\{\!\left(t_{\lambda_{A}},z_{\lambda_{A}}^{\lambda}\right)\!\right\}\right.\!\!,\{(t_{D},D)\}\!\right) \,-\, A_{e}\left(\!\lambda\left|\left\{\!\left(t_{\lambda_{A}},z_{\lambda_{A}}^{\lambda}\right)\!\right\}\right., \{(t_{D}\rightarrow\infty,D)\}\!\right)}{A_{e}\left(\!\lambda\left|\left\{\!\left(\!t_{\lambda_{A}},z_{\lambda_{A}}^{\lambda}\right)\!\right\}\right.\!\!,\{(0,D)\}\!\right) \,-\, A_{e}\left(\!\lambda|\left\{\!\left(t_{\lambda_{A}},z_{\lambda_{A}}^{\lambda}\right)\!\right\},\{(t_{D}\rightarrow\infty,D)\}\!\right)}, \end{aligned}}}  $$

where $A_{e}({\lambda }|\{(t_{\lambda _{A}},z_{\lambda _{A}}^{\lambda })\},\{(t_{D},D)\})$ is the experimental data obtained at wavelength *λ* after period *t*_*D*_ under darkness *D* when the system has been prepared using illumination by $z_{\lambda _{A}}^{\lambda }$ light for duration $t_{\lambda _{A}}$. Here,

$${{\begin{aligned} A\left({\lambda}|\left\{\left(t_{\lambda_{A}},z_{\lambda_{A}}^{\lambda}\right)\right\},\{(0,D)\}\right) &= A\left({\lambda}|\left\{\left(t_{\lambda_{A}},z_{\lambda_{A}}^{\lambda}\right)\right\},\{(t_{D} = 0,D)\}\right),  \\ A\left({\lambda}|\left\{\left(t_{\lambda_{A}},z_{\lambda_{A}}^{\lambda}\right)\right\},\{(t_{D}\rightarrow\infty,D)\}\right) &= {\lim}_{t\to\infty}A\left({\lambda}|\left\{\left(t_{\lambda_{A}},z_{\lambda_{A}}^{\lambda}\right)\right\},\{(t_{D},D)\}\right). \end{aligned}}} $$

Throughout we shall use ${\lim }_{t\to \infty }f(t) = f(t\rightarrow \infty)$. From Eqs. (1) and (2) we can simulate thermal reversion by

$${{\begin{aligned} {A_{t}^{D}} &= \frac{A_{s} \left(\!\lambda\left|\left\{\!\left(\!t_{\lambda_{A}},z_{\lambda_{A}}^{\lambda}\!\right)\!\right\}\right.\!\!,\{(t_{D},D)\}\!\right) \,-\, A_{s}\left(\!\lambda\left|\left\{\!\left(\!t_{\lambda_{A}},z_{\lambda_{A}}^{\lambda}\!\right)\!\right\}\right., \{(t_{D} \! \rightarrow \! \infty,D)\}\!\right)}{A_{s}\left(\!\lambda\left|\left\{\!\left(\!t_{\lambda_{A}},z_{\lambda_{A}}^{\lambda}\!\right)\!\right\}\right.\!\!,\{(0,D)\}\!\right) \,-\, A_{s}\left(\!\lambda|\left\{\!\left(\!t_{\lambda_{A}},z_{\lambda_{A}}^{\lambda}\!\right)\!\right\},\{(t \!_{D}\rightarrow \!\infty,D)\}\!\right)}, \\ &= \frac{c_{\{t_{D},D\}}^{A}-c_{\{t_{D}\rightarrow\infty,D\}}^{A}}{c_{\{0,D\}}^{A}-c_{\{t_{D}\rightarrow\infty,D\}}^{A}}, \end{aligned}}} $$

where $c_{\{t_{D},D\}}^{A} = c^{A}(\boldsymbol {\sigma },\boldsymbol {\Phi },\boldsymbol {\alpha },\boldsymbol {\beta },\{(t_{\lambda _{A}},z_{\lambda _{A}}^{\lambda })\},\{(t_{D},D)\})$ is simplified notation describing the fraction of active photoreceptor, *P*_*A*_, within the molecule population (i.e. in (1) set $c_{\{t_{D},D\}}^{I} = n_{c}-c_{\{t_{D},D\}}^{A}$, the fraction of inactive photoreceptor, *P*_*I*_). Using equation (4) in Section 1.1 of Additional file 1, we get

3 $$ {A_{t}^{D}} = \sum_{j = 1}^{n} \alpha_{j} \mathrm{e}^{-\beta_{j}t_{D}}\ \text{with} \sum_{j=1}^{n} \alpha_{j} = 1,   $$

where *n* is the number of exponentials required to describe the decay of *P*_*A*_. Using (3), we obtain estimates for *α*_*j*_ and *β*_*j*_ (see Section 1.8 of Additional file 1 for pseudo-code). Recall from (1) that the functions *c* ^*k*^ depend on the preparatory light input *Ω*_*P*_. Consequently, the rates of thermal reversion will change depending on the starting fraction of *P*_*A*_ in the system. Importantly, under darkness, Eq. (3) shows that the percentage decrease of active photoreceptor is independent of the measured wavelength, *λ*, used for the analysis since ${E_{t}^{D}}$ is not a function of *λ*. This can be seen in Additional file 1: Figure S1.

### Step 2: Finding search spaces for the transition rates and quantum yield ratio upper bound

The next step of our methodology is to obtain the search spaces for $\sigma _{k}^{\lambda }$ and their respective *Φ*_*k*_. In order to do this, a previously published method will be extended to include the effects of thermal reversion, using the parameters obtained from the previous section [22, 23]. This section will outline the previously published method for photoreceptors with two light-regulated states and extend this to include a single thermal reversion reaction. In the Additional file 1 we present equations for any *n*_*c*_, and cases whereby the photoreceptor undergoes multiple thermal reversion processes or when multiple species exist in the photoreceptor reaction cycle.

Measured spectra are obtained given preparatory conditions $\Omega _{P} = \{{(t_{\lambda _{A}},z^{\lambda }_{\lambda _{A}})\}}$ followed by experimental conditions $\Omega _{E} = \{{(t_{\lambda _{I}},z^{\lambda }_{\lambda _{I}})\}}$ and *vice versa*. Note that, for a given experimental condition $\Omega _{E} = \{(t_{E},z^{\lambda }_{\lambda _{w}})\}$, if *t*_*E*_→*∞* then the resulting spectra do not depend on the preparatory conditions *Ω*_*P*_. Assuming that measured spectra are also related to extinction coefficients via Eq. (1) then we can write:

4 $$ {{\begin{aligned} \hat{\epsilon}_{I}^{\lambda} &= A_{e}\left(\lambda|\Omega_{P},\left\{\left(t\rightarrow\infty,z_{\lambda_{A}}^{\lambda}\right)\right\}\right) \\ &\quad + X_{1}\big(A_{e}\left(\lambda|\Omega_{P},\left\{\left(t\rightarrow\infty,z_{\lambda_{I}}^{\lambda}\right)\right\}\right)-A_{e}\left(\lambda|\Omega_{P},\left\{\left(t\rightarrow\! \infty,z_{\lambda_{A}}^{\lambda}\right)\right\}\right)\!\big), \\ \hat{\epsilon}_{A}^{\lambda} &= A_{e}\left(\lambda|\Omega_{P},\left\{\left(t\rightarrow\infty,z_{\lambda_{A}}^{\lambda}\right)\right\}\right) \\ &\quad + X_{2}\big(A_{e}\left(\lambda|\Omega_{P},\left\{\left(t\rightarrow\infty,z_{\lambda_{I}}^{\lambda}\right)\right\}\right)-A_{e}\left(\lambda|\Omega_{P},\left\{\left(t\rightarrow\! \infty,z_{\lambda_{A}}^{\lambda}\right)\right\}\right)\!\big), \end{aligned}}}  $$

where $\epsilon _{k}^{\lambda }$ was calculated such that $\hat {\epsilon }_{k}^{\lambda } = 2.303{lc}_{tot}{\epsilon }_{k}^{\lambda }$ are the wavelength-dependent extinction coefficients and *X*_*k*_ are scalar constants. Due to the relationship between extinction coefficients, photoconversion cross-sections and quantum yields ($\epsilon _{k}^{\lambda } = \sigma _{k}^{\lambda }/\Phi _{k}$), the resulting spectra provide us with limits for the optimisation algorithm developed below to minimise the difference between our data and Eq. (1).

To simplify our notation we set $c_{\lambda _{w}} = c^{A}(\boldsymbol {\sigma },\boldsymbol {\Phi },\boldsymbol {\alpha },\boldsymbol {\beta },\Omega _{P},\{(t\rightarrow \infty,z^{\lambda _{w}}_{\lambda })\})$ as the fraction of active photoreceptor obtained after infinite exposure to the light-source $z^{\lambda _{w}}_{\lambda }$ given any preparatory conditions *Ω*_*P*_. As such, any absorption spectra can then be defined by

5 $$ {{\begin{aligned} &A_{e}\left(\lambda|\Omega_{P},\left\{\left(t\rightarrow\infty,z^{\lambda}_{\lambda_{w}}\right)\right\}\right) = A_{e}\left(\lambda|\Omega_{P},\left\{\left(t\rightarrow\infty,z^{\lambda}_{\lambda_{A}}\right)\right\}\right)+ \\ &\!\!\left(A_{e}\left(\lambda|\Omega_{P},\left\{\left(t\rightarrow\infty,z^{\lambda}_{\lambda_{I}}\right)\right\}\right)\right. \left. \!\!\!- A_{e}\left(\lambda|\Omega_{P}, \left\{\left(t\rightarrow\infty,z^{\lambda}_{\lambda_{A}}\right)\right\}\right)\right) \!\left(X_{1} + \big(X_{2} - X_{1}\big)c_{\lambda_{w}}\right)\!. \end{aligned}}}  $$

Using Eq. (5), we find that

6 $$\begin{array}{*{20}l} X_{1} &+ (X_{2} - X_{1})c_{\lambda_{A}} = 0,  \\ X_{1} &+ (X_{2} - X_{1})c_{\lambda_{I}}= 1.  \end{array} $$

These equations are implicit for *X*_1_ and *X*_2_ because the fractions $c_{\lambda _{w}}$ depend, via Eq. (4), on unknown $\epsilon _{k}^{\lambda }$ (see Sections 1.2 and 1.3 of Additional file 1). Thus, other than in the simplest cases, these equations require solving numerically. By calculating the values for $c_{\lambda _{w}}$ for given values ***Φ***, one can obtain spectra for $\hat {\epsilon }_{k}^{\lambda }$ via Eqs. (4) and (6). Doing this for all possible values of ***Φ*** provides us with the search space of $\epsilon _{k}^{\lambda }$ spectra to be used within the optimisation algorithm.

The last remaining bound to be obtained is for *R*=*Φ*_*A*_/*Φ*_*I*_. To highlight how the upper bound for *R* can be obtained we look at the case where a photoreceptor has a single thermal reversion reaction. For the case of multiple thermal reversion rates the same steps can be followed to calculate *R*.

For a single thermal reversion rate

$$ c_{\lambda_{A}} = \frac{\sum_{\lambda}\sigma_{I}^{\lambda}z^{\lambda}_{\lambda_{A}}}{\sum_{\lambda}\left(\sigma_{A}^{\lambda}+\sigma_{I}^{\lambda}\right){z^{\lambda}_{\lambda_{A}}}+\beta}   $$

where *β* is the thermal reversion rate in s ^−1^ (for an example see Additional file 1: Figure S1) and the photoconversion cross-sections $\sigma _{k}^{\lambda }$ are in m^2^ mol ^−1^. In our experimental setup, the spectral composition of $z^{\lambda }_{\lambda _{w}}$ follows a Gaussian distribution produced by LED light sources (Additional file 1: Figure S2) [22, 23]. By rearranging Eq. (6) we obtain

7 $$ X_{1} + \big(X_{2}-X_{1}\big)\frac{1}{1+R\frac{\sum_{\lambda}\epsilon_{A}^{\lambda}{z^{\lambda}_{\lambda_{A}}}}{\sum_{\lambda}\epsilon_{I}^{\lambda} {z^{\lambda}_{\lambda_{A}}}}+\frac{\beta}{\Phi_{I}\sum_{\lambda}\epsilon^{\lambda}_{I}{z^{\lambda}_{\lambda_{A}}}}} = 0.  $$

Using Eq. (4) leads to

8 $$\begin{array}{*{20}l} R &= \bigg[\frac{X_{2}}{X_{1}} + \frac{\beta/\Phi_{I}}{{S^{A}_{I}}+X_{1}\big({S^{A}_{A}}-{S^{A}_{I}}\big)}\bigg]\frac{{S^{A}_{I}}+X_{1}\big({S^{A}_{A}}-{S^{A}_{I}}\big)}{X_{2}\big({S^{A}_{I}}-{S^{A}_{A}}\big)-{S^{A}_{I}}}  \\ R &= R^{o} - \frac{\beta/\Phi_{I}}{{S^{A}_{I}}+X_{2}\big({S^{A}_{A}}-{S^{A}_{I}}\big)},  \\ R^{o} &= \frac{X_{2}}{X_{1}}\frac{{S^{A}_{I}}+X_{1}\big({S^{A}_{A}}-{S^{A}_{I}}\big)}{X_{2}\big({S^{A}_{I}}-{S^{A}_{A}}\big)-{S^{A}_{I}}}, \end{array} $$

where ${S_{i}^{j}} = \sum _{\lambda }\epsilon ^{\lambda }_{i}{z^{\lambda }_{\lambda _{j}}}$. The function *R* ^*o*^ is the same as that calculated using the previously published method in the absence of thermal reversion [23]. Note again that the function of *R* is, in fact, implicit. For a given set of ***Φ***, one calculates the values of *X*_*k*_ from Eq. (6) to then obtain a value *R* using Eq. (8). To limit our optimisation search space we wish to find bounds of *R* given our data. By definition, the lower bound of *R* is zero. An upper bound for *R* can be found since the second term of Eq. (8) is always positive since *β*, *Φ*_*I*_ and $\epsilon _{A}^{\lambda }$ are all positive. Thus, *R* ^*o*^ is the upper bound for *R* given specific values of ***Φ***. Therefore, we use the numerical maximum of *R*, *R* ^max^, given all possible combinations of ***Φ*** as the upper bound of *R*.

The above steps therefore provide, in the presence of different numbers of thermal reversion reactions, constraints on the wavelength-dependent photoconversion cross-sections and quantum yields. The final step in obtaining photoconversion cross-sections and quantum yields is to determine where in the defined search space the minimum difference between simulated spectra and experimental data exists.

### Step 3: Simulating time-dependent absorption spectra

In Section 1.7 of Additional file 1 we provide a pseudo-code of this optimization algorithm but, essentially, the problem is to minimize a cost score that describes the difference between the difference spectra obtained from our data and from Eq. (1). The cost score function used in our algorithm calculates the sum of squared residuals such that

9 $$\begin{array}{*{20}l} &\min_{\substack{\Phi_{A}\in[0,1] \\ R\in[0,R^{\text{max}}]}}\chi^{2} = \sum_{t = 1}^{n_{t}}\sum_{\lambda=\lambda_{0}}^{\lambda_{\infty}} \Bigg(\frac{D(t,{\lambda})-S(t,{\lambda})}{\nu_{t}^{\lambda}}\Bigg)^{2}, \\ &\text{with:}  \\ &D(t,{\lambda}) = A_{e}\left({\lambda}\left|\left\{\left(t\rightarrow\infty,z^{\lambda}_{\lambda_{w_{1}}}\right) \right\}\right.,\left\{\left(t,z^{\lambda}_{\lambda_{w_{2}}}\right)\right\}\right)  \\ & \qquad\qquad -A_{e}\left({\lambda}| \left\{\left(t\rightarrow\infty,z^{\lambda}_{\lambda_{w_{1}}}\right)\right\},\left\{\left(0,z^{\lambda}_{\lambda_{w_{2}}}\right)\right\}\right),  \\ & S(t,{\lambda}) = A_{s}\left({\lambda}\left|\left\{\left(t\rightarrow\infty,z^{\lambda}_{\lambda_{w_{1}}}\right)\right\}\right.,\left\{\left(t,z^{\lambda}_{\lambda_{w_{2}}}\right)\right\}\right)  \\ & \qquad\qquad -A_{s}\left({\lambda}\left| \left\{\left(t\rightarrow\infty,z^{\lambda}_{\lambda_{w_{1}}}\right)\right\}\right.,\left\{\left(0,z^{\lambda}_{\lambda_{w_{2}}}\right)\right\}\right),  \end{array} $$

where $\nu _{t}^{\lambda }$ is the experimental error of our data, $A_{e}({\lambda }|\{(t\rightarrow \infty,z^{\lambda }_{\lambda _{w_{1}}})\},\{(t,z^{\lambda }_{\lambda _{w_{2}}})\})$, measured between wavelengths *λ*∈[*λ*_0_,*λ*_*∞*_]. Here, $z^{\lambda }_{\lambda _{w_{1}}}$ and $z^{\lambda }_{\lambda _{w_{2}}}$ are the preparatory and experimental light conditions, respectively, that can be represented by Gaussian distributions centred at $\lambda _{w_{1}}$ and $\lambda _{w_{2}}$. Furthermore, *n*_*t*_ is the number of measured time-points (in our case *n*_*t*_=5) [42]. In the case where simulated data is used without noise, we set $\nu _{t}^{\lambda } = 1$. Thus, upon estimating a value of *Φ*_*A*_ and *R*:

- One obtains a value of *Φ*_*I*_ from *R*=*Φ*_*A*_/*Φ*_*I*_.
- These values are then input into Eq. (6) to obtain values of *X*_1_ and *X*_2_.
- The obtained values of *X*_1_ and *X*_2_ are used in Eqs. (4) and (5) to obtain $\epsilon _{A}^{\lambda }$ and $\epsilon _{I}^{\lambda }$.
- These spectra, plus the estimates of ***Φ***, are input into Eq. (1) to simulate absorption spectra $A_{s}\left ({\lambda }\left |\left \{\left (t_{D}\right.\rightarrow \infty,z^{\lambda }_{\lambda _{w_{1}}}\right)\right \},\left \{\left (t,z^{\lambda }_{\lambda _{w_{2}}}\right)\right \}\right)$.

In our study all optimisation routines use MATLAB’s *fmincon* function that is started from 100 random initial points to cover as much of the global parameter space as possible. Furthermore, 95% confidence intervals for *Φ*_*k*_ and thermal reversion parameters are calculated from profile likelihoods following the method outlined in [42].

### Software

All computational procedures were performed using MATLAB (Mathworks, Massachusetts, USA). The codes created for our optimisation algorithms can be downloaded from http://gitlab.com/wurssb/Absorption_Spectra_Optimization.

## Results

### Methodology successfully returns input values for extreme simulated test cases

To test the validity of our algorithm, we have tested the system using simulated data and compared the output to results obtained using Butler’s model [11]. Here, we highlight two extreme test cases in the absence of noise. Notably, we assume that the light-dependent switching of the photoreceptor population occur on the ms to s timescale, comparable to what is known for full-length plant phytochromes in vivo (see Additional file 1: Figure S3) [13]. For full details of the parameters used in our test cases see Additional file 1: Tables S1 and S2.

**Fig. 2:**
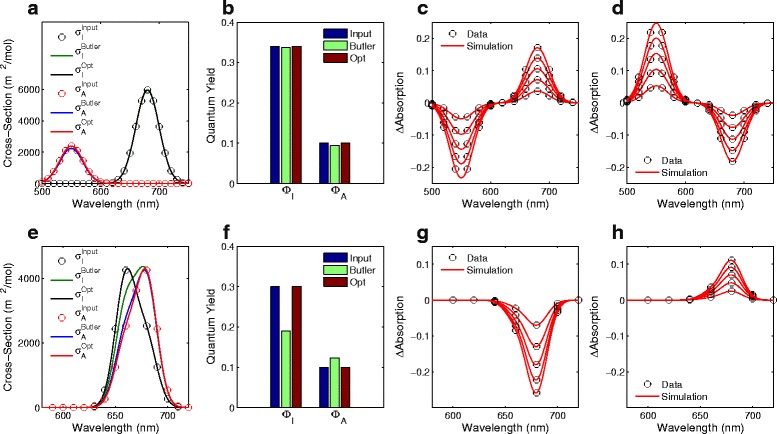
Test cases of photoreceptors against output from Butler’s model. **a**–**d** A red/green photoreceptor that satisfies the assumptions required for the photoconversion cross-sections to be calculated using Butler’s method. **a** Circles are the target photoconversion cross-sections used to create the synthetic data (denoted ‘Input’); blue and green lines show the photoconversion cross-sections calculated using Butler’s model (denoted ‘Butler’); black and red lines show the photoconversion cross-sections obtained using our optimisation algorithm (denoted ‘Opt’). **b** Quantum yields obtained from Butler’s model (denoted ‘Butler’) and our optimisation algorithm (‘Opt’) compared to the target values (‘Input’). **c**, **d** Simulations of absorption spectra (*red lines*) created using the optimal values from our algorithm compared to synthetic datasets (*circles*). **e**–**h** Same as in **a**–**d** except for a photoreceptor with photoconversion cross-sections that do not satisfy the conditions required to be calculated by Butler’s model

In the first test case, we simulated a red/green cyanobacteriochrome [4, 25]. The photoconversion cross-sections of this photoreceptor do not overlap in the wavelength-domain and we set the thermal reversion reaction rate to zero. Thus, under red or green illumination conditions (660 nm or 550 nm, respectively) the photoreceptor is able to attain populations containing 100% active or inactive photoreceptor. Furthermore, we defined the quantum yields to be *Φ*_*A*_=0.1 and *Φ*_*I*_=0.34, which are broadly reasonable values for cyanobacteriachromes [25]. As shown in Fig. 2 a, our algorithm is able to find the input wavelength-dependent photoconversion cross-sections and the correct quantum yields (Fig. 2 b). Furthermore, since the experimental conditions can lead to populations with 100% *P*_*I*_, Butler’s model provides similar results (Fig. 2 a, b). Consequently, the optimal simulations directly overlap with the simulated test data (Fig. 2 c, d).

To highlight that our algorithm is an accurate generalisation of Butler’s model, we now show the results for a photoreceptor that does not satisfy the conditions required for Butler’s model to produce accurate results. In this test case, we have simulated a photoreceptor that is activated by 660 nm light, but inactivated by 680 nm light with quantum yields of *Φ*_*A*_=0.1 and *Φ*_*I*_=0.3. Such a case could occur when analysing point mutations within phytochromes (see [35] for examples). Since this photoreceptor is unable to obtain populations containing 100% of *P*_*I*_ or *P*_*A*_, Butler’s model fails to predict the correct photoconversion cross-sections and quantum yields (blue and green lines in Fig. 2 e, f). However, our algorithm finds the correct photoreceptor parameters and simulations perfectly match the synthetic data (Fig. 2 e–h). Thus, we have shown that, in both cases where Butler’s model can be applied and where it cannot, our optimisation algorithm is able to find the ‘true’ photoconversion cross-sections and quantum yields of photoreceptors.

### Approach is able to find optimal photoconversion cross-sections in the presence of multiple biochemical reactions

As discussed in Section 1.1 in Additional file 1, there is more than one way in which a photoreceptor can undergo multiple thermal reversion reactions. One option is through photoreceptor-chromophore interactions crucial for the transformation from active to inactive photoreceptor. The second is through dimerisation, whereby the *P*_*A*_−*P*_*I*_ hetero-dimer has a different thermal reversion rate to the *P*_*A*_−*P*_*A*_ homo-dimer, as is the case for full-length phyB found in plants [28]. Notably, if spectra are obtained from a sample that contain a mix of these populations, we still wish to obtain photoconversion cross-sections for the monomeric *P*_*A*_ and *P*_*I*_ species. To show that our methodology can handle both cases, we have simulated a red/far-red photoreceptor using the ‘Mancinelli’ spectra and quantum yields in the presence of multiple thermal reversion reactions [13].

**Fig. 3:**
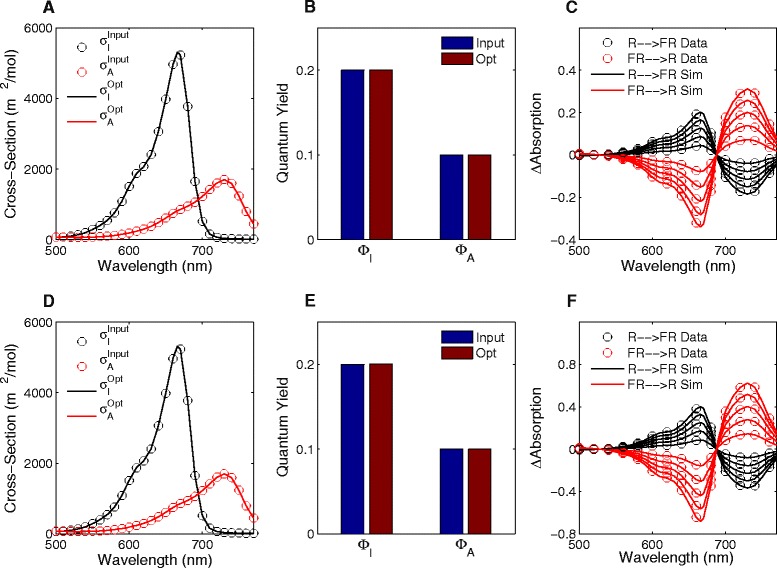
Optimisation results for photoreceptors undergoing multiple thermal reactions. **a**–**c** A red/far-red photoreceptor undergoing multiple thermal reversion reactions due to photoreceptor-chromophore interactions. **a** Comparison of optimal photoconversion cross-sections (‘Opt’) with those used as input (‘Input’). **b** Quantum yields obtained using algorithm (‘Opt’) compared with those used to create the synthetic data (‘Input’). **c** Comparison of synthetic data (*circles*) compared with simulations obtained using optimal parameters (*lines*) for our red-to-far-red (*black*) and far-red-to-red (*red*) experiments. **d**–**f** Same as in **a**–**c** for a red/far-red photoreceptor undergoing two thermal reversion rates due to dimerisation

Figure 3 a–c shows the results for the red/far-red photoreceptor undergoing multiple thermal reversion due to photoreceptor-chromophore interactions. We set 70% of the active phytochromes to undergo one thermal reversion reaction rate (*β*_1_ = 0.005 s ^−1^) whilst the other 30% undergo a reaction rate an order of magnitude smaller (*β*_2_ = 0.0005 s ^−1^). We are, once again, able to find the correct photoconversion cross-sections and quantum yields that produced the synthetic data (Fig. 3 a, b). Simulations using these parameters thus show a complete match with the input data (Fig. 3 c).

By changing the *c* ^*k*^ function, we are able to describe absorption spectra that result from mixed populations of dimerised photoreceptors. Again, using an order of magnitude difference between the thermal reversion rates, we find the parameters used to create our test data (Fig. 3 d, e). The resulting simulations using these parameters match the synthetic datasets (Fig. 3 f). Furthermore, we can find the optimal photoconversion cross-sections for any given set of thermal reversion parameters and using the wrong form of *c* ^*k*^ has negative effects on results (Additional file 1: Figure S5).Thus, we have shown that even in complex photoreceptor systems our approach is able to accurately find the biochemical parameters of population-level state transitions for photoreceptors.

### Performance is robust to noisy spectra

**Fig. 4:**
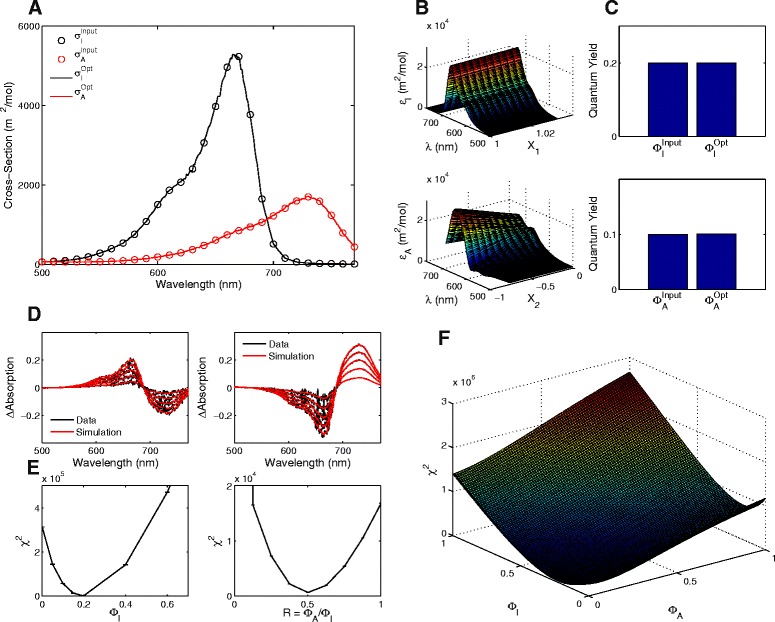
Approach can obtain input values in the presence of 10% noise. **a** Optimal photoconversion cross-sections (lines, denoted ‘Opt’) compared to the input photoconversion cross-sections used to create the test data (*circles*, denoted ‘Input’). **b** Search spaces calculated for the extinction coefficients using the Verméglio method as a function of the scalar constants, *X*_1_ and *X*_2_. **c** Comparison of optimal quantum yields (denoted ‘Opt’) compared to the target values (denoted ‘Input’). **d** Simulated absorption spectra (*red lines*) compared to the synthetic data (*black lines*). For the time-points of each measurement see Additional file 1: Table S1. **e** Profile likelihoods for the parameters obtained from our optimisation algorithm [42]. **f** The search space for the optimal quantum yields. The space shows a clear global minimum at the optimal values

In the last two sections we presented results in the absence of any experimental noise. However, as with all experiments, data is likely to include noise from both external and biological sources. Consequently, the methodology should be robust against noise and provide accurate estimates of the photoreceptor transition rates. By including stochastic fluctuations (with coefficient of variation = 10%) on simulated data from a single-step thermal reversion red/far-red photoreceptor (thermal reversion rate *β* = 0.005 s ^−1^), we are able to compare the impact of noise on our simulation results. As seen in Fig. 4, we are able to find the input photoconversion cross-sections from the calculated search spaces (Fig. 4 a, b). Similarly, the quantum yield estimates are accurate in the presence of noise (Fig. 4 c). The resulting simulations using the optimal parameters also match the noise-inclusive data (Fig. 4 d).

Due to the inclusion of noise, we also highlight here the accuracy of our approach to find the optimal parameters. In Fig. 4 e, we show the profile likelihoods obtained for *Φ*_*I*_ and *R*=*Φ*_*A*_/*Φ*_*I*_. Following the theory in [42], the 95% confidence interval for these parameters occurs when changing either parameter leads to the optimal score increasing by more than *χ* ^2^(0.95,1)=3.8415. From Fig. 4 e, it is clear to see that a very minor change in either parameter would result in increasing the score by more than 3.8415 (note the scale of the y-axis), suggesting that our methodology obtained the optimal parameter values with high confidence. In Fig. 4 f, we show the entire search space for our optimisation algorithm. The search space shows a clear minima at [ *Φ*_*A*_,*Φ*_*I*_]=[ 0.1,0.2] that our algorithm has successfully found.

On the basis of these results we can confirm that our approach provides accurate results for multiple photoreceptors undergoing different thermal reversion reactions in the presence and absence of noise. Furthermore, the obtained parameters have been shown to be the global minimum of the search space. This would suggest that our methodology is well-suited to dealing with absorption spectra obtained experimentally. In the following sections the transition rates for the phyB-N protein fragment commonly used in optogenetic tools will be determined.

### The phyB-N protein fragment undergoes slow thermal relaxation

The N-terminal region of phyB (phyB-N) contains the key protein domains required for light responses and is relatively stable compared to other phyB fragments upon purification [9]. Consequently, this protein is ideal for optogenetic tools although the kinetic rates describing the inter-protein reactions have not yet been quantified. Before detailing the photoconversion cross-sections that control the speed of light-dependent reaction, in accordance with our methodology, we need to determine the reaction rates of thermal reactions of phyB-N.

**Fig. 5:**
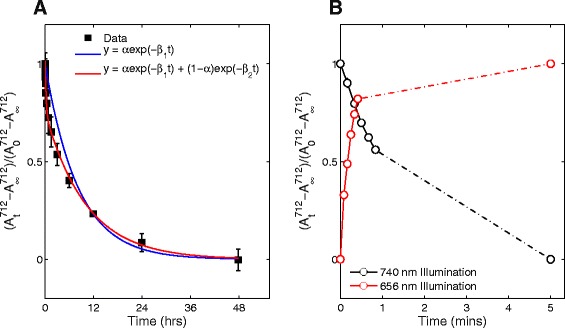
Population of phyB-N molecules responds to light and darkness on the minute to hour time-scale. **a** Thermal reversion kinetics of phyB-N (*black squares*) compared to optimal function for a single (*blue line*) and bi-exponential decay function (*red line*). **b** Production and decay of *P*_*A*_ during red light illumination (*red line*) and far-red illumination (*black line*). *Dashed line* shows the linear trend between the final measured time-point and the starting conditions of the reverse experiment obtained after 5 min illumination. Experimental *curves* obtained using (2)

Before conducting our absorption experiments we confirmed the presence of phyB-N (∼70 kDa) within our sample (Additional file 1: Figure S4). The sample was placed under 11 *μ*mol m ^−2^ s ^−1^ red light until the system had saturated to provide the highest possible amount of active photoreceptor within the population (see Section 2 of Additional file 1). Over the next 48 hours, absorption spectra were recorded in a temperature controlled dark chamber. As observed in Fig. 5 a, the percentage of active phyB-N reaches a minimum by the end of the 48h protocol, showing levels of absorption at 712 nm that are similar to those seen after a saturating level of far-red light. Comparatively with this slow thermal relaxation, the light-regulated changes of photoreceptor populations occurs within minutes given the light conditions and intensities (11 *μ*mol m ^−2^ s ^−1^) used in our study (Fig. 5 b). From this result, one should note two observations. First, that as light intensity increases (decreases) the speed of conformational change will also increase (decrease). Second, given that we use the same intensity of red and far-red light in our experiments, Fig. 5 b suggests that the activation of phyB-N molecule population by red light is relatively faster than deactivation by far-red light.

To determine the rate of thermal reversion, we used our optimisation algorithm to fit exponential functions to the data (see ‘Methods’ above). By fitting a single and double exponential function to the data (blue and red line in Fig. 5 a, respectively), we found that the data could be described qualitatively well by both single and double thermal reversion rates. The biochemical cause of the second exponential rate within the thermal reversion reactions is not well understood, however we have hypothesised potential causes in Section 1.1 of Additional file 1. Since the focus of the work is on obtaining the photoreceptors photoconversion cross-sections, we analyze the generally accepted case of a single thermal reversion reaction. The optimal value for the parameter was *β*=3.4×10^−5^ s ^−1^ (see Table 1 for full set of estimated rates and confidence intervals). Compared to published data for this protein, this is a slower thermal reversion rate than that recorded in [35]. However, and arguably most importantly, the thermal reversion of phyB-N in vitro is slower than thermal reversion of full-length phytochrome in vivo as has been observed previously [28, 35, 43].

**Table 1:** Optimal thermal reversion parameters with 95% confidence intervals

	Single exponential	Double exponential
*α*	1	0.265 ± 0.0094
*β*_1_	3.4 ×10^−5^± 6 ×10^−10^	0.0011 ± 0.0011
*β*_2_	0	2.7 ×10^−5^± 9 ×10^−6^

Confidence intervals calculated from profile likelihoods [42]

### Conformational changes of phyB-N are slower and blue-shifted compared to full-length phyB

According to the ‘Mancinelli spectra’ for full-length phytochromes, the speed of transitioning between a population of active and inactive photoreceptor would occur within 5-20 s under the experimental conditions of this work (see Section 2 of Additional file 1 and Additional file 1: Figure S3) [13]. However, there are two important differences in conditions between the measurement of the ‘Mancinelli spectra’ and our experimental conditions. First, the ‘Mancinelli spectra’ were calculated using absorption spectra obtained from in vivo samples whereas the samples used here are in vitro. Second, in plant systems, phytochromes use P *Φ*B chromophores whereas, in synthetic systems, PCB is used as an alternative. Such differences have already been shown to produce ∼20 nm blue-shifted absorption spectra that transition more slowly between different states (∼mins) than spectra measured in vivo (∼s) [35, 44]. Our data support these observations, whereby saturating levels of active or inactive photoreceptor can be achieved within 5 min given our experimental conditions (Fig. 5 b).

**Fig. 6:**
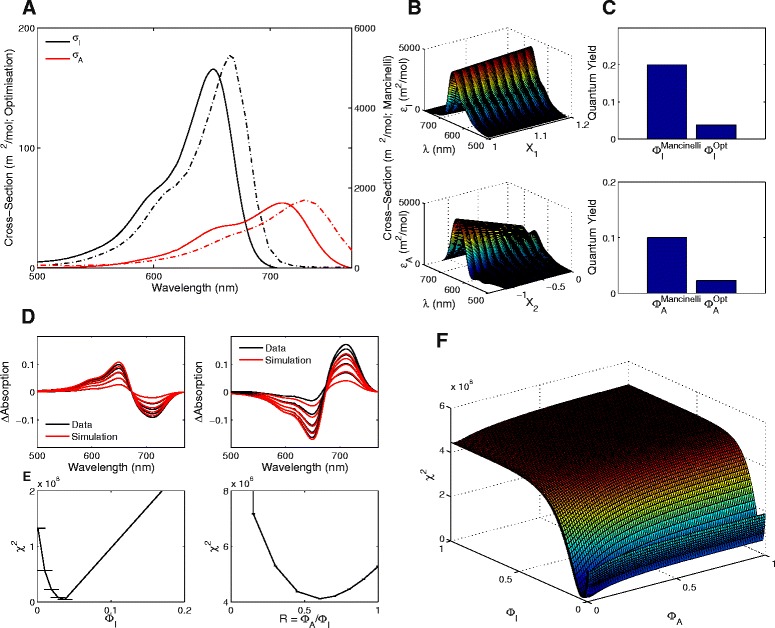
Optimal photoconversion cross-sections obtained for phyB-N. **a** Optimal photoconversion cross-sections (solid lines) compared to the ‘Mancinelli spectra’ (*dashed* lines) calculated for full-length phytochromes. Note the change in y-axis. **b** Search spaces calculated for the extinction coefficients using the Vermémeglio method as a function of *X*_1_ and *X*_2_. **c** Comparison of optimal quantum yields (denoted ‘Opt’) compared to the ‘Mancinelli quantum yields’ (denoted ‘Mancinelli’). **d** Simulated absorption spectra (*red* lines) compared to the synthetic data (*black* lines). **e** Profile likelihoods for the parameters obtained from our optimisation algorithm. **f** The search space for the optimal quantum yields

Using these results and the thermal reversion rates obtained in the last section, we are now in a position to use our optimisation algorithm to find the optimal photoconversion cross-sections and quantum yields for phyB-N (Fig. 6). The output of our algorithm provides three notable observations. First, as would be expected by the slower kinetics, the amplitude of the resulting photoconversion cross-sections (that are proportional to light-regulated kinetic rates) is much lower than those obtained for full-length phytochromes in vivo (Fig. 6 a). Second, the peak of the photoconversion cross-section for phyB-N is blue-shifted compared to full-length phytochromes with peaks occurring ∼20 nm shorter than full-length phytochromes in a similar manner to absorption spectra measured from in vivo and in vitro samples (Fig. 6 a). Third, in accordance with the slower kinetic rates and lower amplitude photoconversion cross-sections of phyB-N, the quantum yields are similarly decreased to a smaller percentage than previous estimates of full-length and mutated phytochromes, with *Φ*_*A*_=0.023±0.0014 and *Φ*_*I*_=0.038±0.0034 (Fig. 6 c). In comparison to obtaining the results using Butler’s model, we found that the calculated photoconversion cross-sections are similarly blue-shifted to our calculations albeit the amplitude of the rates and the quantum yields are decreased (to below 1%) in comparison to our estimates (see Additional file 1: Figure S6). Our results suggest that the population level responses of phyB-N, in our hands, respond slowly to changes in light condition. However, despite these differences in kinetics with full-length phytochromes, the optimal parameters produce simulations that match closely with the experimental data (Fig. 6 d). Furthermore, given the profile likelihoods and the calculated search space, the algorithm indicates that the global optimum set of parameters has been found (Fig. 6 e, f). Since we have shown that our approach can handle data that contains noise, the differences observed between the data and our optimal simulations can be attributed to external sources that are not currently accounted for within our model (see ‘Discussion’ below).

## Discussion

### Methodology accurately calculates properties of photoreceptors

In this work we have introduced and tested a methodology that calculates the wavelength-dependent photoconversion cross-sections and the quantum yield of photoreceptor states (Fig. 1). Based on theoretical analysis (see [11, 15, 23]) of specific photoreceptor conditions, we have generalised the theory to be used for any studied photoreceptor (see Sections 1.4 and 1.5 of Additional file 1). The key step in the algorithm is the construction of search spaces for the *k* species within a photocycle. These search spaces contain all possible sets of photoconversion cross-sections, $\sigma _{k}^{\lambda }$, given different values of quantum yields, *Φ*_*k*_ (for example, see Figs. 4 and 6 b). The construction of these search spaces involves solving simultaneous equations that depend on the fraction of active photoreceptor (Eqs. (4) and (6)). Thus, given the constraints of Eq. (4), the wavelength-dependency of the photoconversion cross-sections can have no other form (i.e. the solution to the optimisation problem definitely exists within this space). Consequently, we have demonstrated that the approach works for different photoreceptors with multiple thermal reversion reactions and dimer forms in the presence or absence of intrinsic noise (Figs. 2, 3 and 4). In Section 1.4 and 1.5 of Additional file 1, we have presented the equations and an outline of how our analysis could be adapted for further photoreceptors that respond only to a single wavelength of light (i.e. for blue light receptors such as cryptochromes and LOV-based proteins that are used in optogenetics [45]) or for photoreceptors that have reaction cycles containing *N* species.

The approach presented here has high potential to be used to obtain the photoconversion cross-sections for these photoreceptors, aiding both plant and synthetic biologists in understanding their respective light responsive networks in greater detail. One such example where our algorithm can be used is for the plant UV-B photoreceptor, UVR8 (UV RESISTANCE LOCUS 8), that has recently been shown to exist as a mixed population of active monomers and inactive dimers under different environmental conditions [46]. Our methodology will be instructive in determining photoconversion cross-sections for this photoreceptor and the rates of this light-activated switch. Furthermore, since our proposed method can handle cases of overlapping photoconversion cross-sections more robustly than previous methods, one can envisage that it can be used to obtain transition rates for photoreceptors containing point mutations (as in [35]) or to find photoconversion cross-sections of photoreceptor sub-states, such as Lumi-R in the case of phytochromes [19–21]. In Section 1.5 of Additional file 1 we highlight how this analysis could be achieved, however it should be noted that obtaining analytical expressions of *c* ^*k*^ and *R* becomes challenging as the number of system components increases. Thus, one could envisage that numerical solves are incorporated into the analysis to approximate values of *c* ^*k*^ given a specific model system.

As with all methodologies, accuracy of the results is one of two factors that need to be taken into account. Whilst we have shown that our approach produces accurate results, we should also assess computational performance. Currently, running the entire process in a sequential manner on a single iMac core using MATLAB R2013a can take at least a couple of hours when 100 optimisation runs are performed, depending on the complexity of the photoreceptor system being studied. However, within the current framework there are many steps that could be parallelised. Notably, the most time-consuming step is generating the transition rate search spaces, which becomes particularly inefficient for complex problems (such as phytochrome dimers). Upon parallelisation, this process could be sped up since the same calculations are repeatedly performed for different values of the quantum yields. Furthermore, our optimisation procedure is currently performed 100 times starting from random initial places with the quantum yield parameter space. This could similarly be parallelised such that all optimisation runs take place simultaneously. One could envisage that these steps would decrease the computational speed from hours to minutes.

### The phyB-N protein fragment used in optogenetic tools responds slowly to changing light conditions

To showcase how our approach can be applied to a well-studied photoreceptor, we performed our analysis on the phyB N-terminal protein fragment that is used for optogenetic tools [26, 47]. Our results highlight three important insights. First, as previously observed, the data show that the phyB-N fragment has very slow thermal reversion kinetics and light-reversibility (Fig. 5 a, b) compared to other phytochrome protein variants (for comparison, see the predicted in vivo light-reversibility of full-length phytochromes predicted from the photoconversion cross-sections in Additional file 1: Figure S3) [13, 28, 35, 43]. Second, the calculated photoconversion cross-sections have a lower amplitude for phyB-N in vitro than full-length phytochromes in vivo and have a wavelength-dependency that is relatively blue-shifted (Fig. 6 a). This matches with previous observations of measured *P*_*A*_ accumulation and blue-shifted absorption spectra of phyB-N [9, 35]. Finally, in correlation with the slower transition between *P*_*A*_ and *P*_*I*_, the efficiency of the phyB-N protein to absorb photons (as determined by the quantum yields *Φ*_*A*_ and *Φ*_*I*_) are decreased compared to estimates for full-length phytochromes [13, 25]. This is likely due to the different chromophores used by phytochromes under in vivo and in vitro conditions [44]. In summation, these results suggest that responses of phyB-N populations in vitro to light are inefficient and slow when compared to the full-length counterpart in vivo.

This result has implications for the development of optogenetic tools based on the light-switching of phyB-N [26, 27, 47]. These studies have shown that common readouts of phyB-based optogenetic tools, such as gene expression, can take hours to accumulate to high levels after illumination of red light. Notably, naturally occurring processes such as nuclear transport are rate-limiting and can take 20 min before the maximal system response is observed [27]. Since the phyB-N population is fully activated by red light within 5 min of our experimental set up (using 11 *μ*mol m ^−2^ s ^−1^ LEDs), our results suggest that accelerating population level switches between photoreceptor states could also, consequently, increase the speed of optogenetic responses. For example, mutated forms of phyB-N that have faster activation kinetics (such as the 90–624 amino acid fragment [35]) could be applied in optogenetic tools.

## Conclusion

Here, we have built upon previous theoretical work to present a generalised approach for determining photoconversion cross-sections and, consequently, transition rates between conformational states of photoreceptors. We have shown the utility of our approach through the use of simulated data for multiple photoreceptors of differing complexity before performing our analysis on the phyB N-terminal fragment used in optogenetic tools. We envisage that the methodology can be used for any photoreceptor protein fragment, allowing the research field to directly compare rates at which photoreceptors and their mutated derivatives switch between conformational states in more detail. The resulting analysis will aid future optogenetic efforts where speed, efficiency and robustness of photoreceptor dynamics are imperative for the application of tools in bio-based industries.

## Additional file

Additional file 1 Appendix. Appendix contains: the mathematical details to calculate the percentage of active photoreceptor within the population; calculating different forms of steady-state *P*_*A*_ percentages; how the *P*_*A*_ percentage is related to the scalar values *X*_1_ and *X*_2_ used in the Verméglio method; how the approach could be applied to simpler and more complex photoreceptor systems; using Butler’s model; pseudo-code for the optimisations algorithms used; experimental methods; Supplementary Figures and Tables. (PDF 290 kb)
