# Supplementary material for: Unearthing the transition rates between photoreceptor conformers
Source: BMC Syst Biol. 2016 Nov 25;10:110. doi: 10.1186/s12918-016-0368-y (PMC5123409; doi:10.1186/s12918-016-0368-y)
Supplement: Additional file 1 — Appendix. Appendix contains: the mathematical details to calculate the percentage of active photoreceptor within the population; calculating different forms of steady-state P A percentages; how the P A percentage is related to the scalar values X 1 and X 2 used in the Verméglio method; how the approach could be applied to simpler and more complex photoreceptor systems; using Butler’s model; pseudo-code for the optimisations algorithms used; experimental methods; Supplementary Figures and Tables. (PDF 290 kb) [file 12918_2016_368_MOESM1_ESM.pdf]

Unearthing the transition rates between photoreceptor conformers: Appendix

Robert W. Smith[*] [†] Britta Helwig[*] [‡], Adrie H. Westphal[‡], Eran Pel[*] [‡],
Maximilian Hörner[§] [¶], Hannes M. Beyer[§] [¶], Sophia L. Samodelov[¶] [‖], Wilfried Weber[§],
Matias D. Zurbriggen[‖], Jan Willem Borst[‡], Christian Fleck[*] [**]

Contents

1 Additional Computational Methods **2**
1.1 A mathematical model for phytochrome undergoing two thermal reversion processes . . . 2
1.2 Forms of steady-state percentages of active photoreceptor, c_{λ_w} . . . 5
1.3 Relating c's to X's in Verméglio method . . . 7
1.4 Application to a simpler photoreceptor system . . . 9
1.5 Generalising method to photocycles containing N species . . . 10
1.5.1 Example: the three-species cycle of phytochromes . . . 12
1.6 Calculation of phyB-N photoconversion cross-sections using Butler's method . . . 14
1.7 Absorption spectra optimisation algorithm . . . 16
1.8 Thermal reversion optimisation algorithm . . . 18

[*]Laboratory of Systems & Synthetic Biology, Wageningen UR, PO Box 8033, 6700 EJ Wageningen, The Netherlands.
[†]LifeGlimmer GmbH, 12163 Berlin, Germany
[‡]Laboratory of Biochemistry, Wageningen UR, PO Box 8128, 6700 ET Wageningen, The Netherlands.
[§]Faculty of Biology & BioSS, University of Freiburg, 79104 Freiburg, Germany
[¶]Spemann Graduate School of Biology, University of Freiburg, 79104 Freiburg, Germany
[‖]Institute of Synthetic Biology, Heinrich Heine University, 40225 Düsseldorf, Germany
[**]Corresponding author: christian.fleck@wur.nl.

2 **Experimental Methods** **18**
2.1 Protein purification 18
2.2 Protein analysis protocol 19
2.3 Absorption spectra measurements of photoswitching 19
2.4 Thermal reversion experiments 20

3 **References** **22**

4 **Supplementary Tables** **23**

5 **Supplementary Figures** **27**

1 Additional Computational Methods

1.1 A mathematical model for phytochrome undergoing two thermal reversion processes

In the main text, we have shown a number of examples highlighting the utility of our methodology. Notably, cases whereby a population of photoreceptors contains two sub-populations undergoing different thermal reversion rates. One could envisage that this is due to multiple reactions within a monomeric photoreceptor or that different conformers of photoreceptor dimers undergo different thermal reversion reactions as is the case for full-length phyB from *Arabidopsis* [1]. Since the case of photoreceptor dimerisation has been treated mathematically elsewhere (see [1] and Section 1.2), we present a model whereby a monomeric photoreceptor undergoes multiple thermal reversion reactions.

The model reads

$$\begin{aligned}
\dot{P_I} &= k_2 P_A + \beta_1 P_A + \delta C_I - (k_1 + \gamma H) P_I \\
\dot{P_A} &= k_1 P_I + \delta C_A - (k_2 + \beta_1 + \gamma H) P_A \\
\dot{C_I} &= k_2 C_A + \gamma H P_I + \beta_2 C_A - (k_1 + \delta) C_I \\
\dot{C_A} &= k_1 C_I + \gamma H P_A - (k_2 + \delta + \beta_2) C_A \\
\dot{H} &= \delta (C_I + C_A) - \gamma H (P_I + P_A),
\end{aligned} \tag{1}$$

where k_1 and k_2 are the light induced reactions that are proportional to the photoconversion cross-sections ($k_i = \sum_\lambda \sigma_i^\lambda z_{\lambda_w}^\lambda$ as described in [2–4] and z_{λ_w} is the photon distribution from the light source as described in the main text), β_1 and β_2 are the two thermal reversion rates, γ is the rate of complex association and δ is the rate of dissociation. Note that synthesis and degradation rates have been ignored, since these processes do not occur within our photoreceptor samples that are purified

and measured in solution. Here P is assumed to be 'free' photoreceptor molecules, whereas C could take one of two interpretations. First, C is some form of complexed photoreceptor with some unknown interaction partner, H. For example, in our system phyB-N is thought to exist as a monomer. Therefore, H must either be very small or is intrinsically part of the light reaction mechanisms of phyB-N, which could be the case for light-sensing chromophores [5,6]. In our model, C could represent a phyB-chromophore complex that alters the dynamics between conformational states. On the other hand, P and C could be distinct sub-populations of photoreceptor proteins whereby the protein folding machinery produces distinct isomers. For example, dimerization within the protein structure of phyB is known to occur through the GAF domain [7,8]. Thus, P could be one form of phytochrome whereby the GAF domains are unbound, but C represents a sub-population where GAF domains are bound, altering conformational switches. The aim of our work in this section is to solve this system to provide a general form of the equation that describes P_A decay in the presence of light and thermal reactions.

The total amount of phytochrome in the system is $P_0 = P_I + P_A + C_I + C_A$ and the total amount of H, $H_0 = H + C_I + C_A$, is conserved. Therefore, by setting $\dot{X} = \dot{P_I} + \dot{P_A}$ and $\dot{Y} = \dot{C_I} + \dot{C_A}$ one can find that

$$
\begin{aligned}
X_{ss} &= P_0 - Y_{ss}, \\
H_{ss} &= H_0 - Y_{ss}, \\
Y_{ss} &= \frac{1}{2}\left[\frac{\beta}{\gamma} + H_0 + P_0 - \sqrt{\left(H_0 + P_0 + \frac{\beta}{\gamma}\right)^2 - 4H_0P_0}\right],
\end{aligned}
$$

where $0 \leq Y_{ss} \leq P_0$. Using these results we can then re-write our system in terms of P_A and $Z = P_A + C_A$

$$
\begin{aligned}
\dot{P_A} &= k_1(X_{ss} - P_A) + \delta(Z - P_A) - (k_2 + \beta_1 + \gamma H_{ss})W \\
\dot{Z} &= k_1(P_0 - Z) - (\beta_1 - \beta_2)P_A - (k_2 + \beta_2)Z.
\end{aligned}
$$

We solve the inhomogeneous equation

$$
\begin{aligned}
\dot{u} &= Au + b, \\
u &= (P_A, Z)^T,
\end{aligned}
$$

for $u_{ij} = v_{ij}\mathrm{e}^{\psi_j t}$. The resulting eigenvalues and eigenvectors are

$$\rho_{\pm} = -(k_1 + k_2) - \frac{1}{2}\left[\gamma H_{ss} + \delta + \beta_1 + \beta_2 \pm \sqrt{(\gamma H_{ss} + \delta + \beta_2 - \beta_1)^2 - 4\delta(\beta_2 - \beta_1)}\right] \tag{2}$$

$$v = \begin{pmatrix} \frac{\gamma H_{ss}+\delta+\beta_2-\beta_1+\sqrt{(\gamma H_{ss}+\delta+\beta_2-\beta_1)^2-4\delta(\beta_2-\beta_1)}}{2(\beta_2-\beta_1)} & \frac{\gamma H_{ss}+\delta+\beta_2-\beta_1-\sqrt{(\gamma H_{ss}+\delta+\beta_2-\beta_1)^2-4\delta(\beta_2-\beta_1)}}{2(\beta_2-\beta_1)} \\ 1 & 1 \end{pmatrix}.$$

Using the initial condition

$$u(0) = \left(\frac{v_{12}Z(0) - v_{22}P_A(0)}{v_{12}v_{21} - v_{11}v_{22}}, \frac{v_{11}Z(0) - v_{21}P_A(0)}{v_{12}v_{21} - v_{11}v_{22}}\right),$$

the general solution is

$$u(t) = \begin{pmatrix} u_1(0)v_{11}e^{\rho_+ t} - u_2(0)v_{12}e^{\rho_- t} + \frac{v_{11}(v_{12}b_2 - v_{22}b_1)}{\rho_+(v_{11}v_{22} - v_{12}v_{21})}(1 - e^{\rho_+ t}) + \frac{v_{12}(v_{21}b_1 - v_{11}b_2)}{\rho_-(v_{11}v_{22} - v_{12}v_{21})}(1 - e^{\rho_- t}) \\ u_1(0)v_{21}e^{\rho_+ t} - u_2(0)v_{22}e^{\rho_- t} + \frac{v_{21}(v_{12}b_2 - v_{22}b_1)}{\rho_+(v_{11}v_{22} - v_{12}v_{21})}(1 - e^{\rho_+ t}) + \frac{v_{22}(v_{21}b_1 - v_{11}b_2)}{\rho_-(v_{11}v_{22} - v_{12}v_{21})}(1 - e^{\rho_- t}) \end{pmatrix}.$$

For $Z(t) = P_A(t) + C_A(t)$, we find

$$Z(t) = Ae^{\rho_+ t} + Be^{\rho_- t} + C\left(1 - e^{\rho_+ t}\right) + D\left(1 - e^{\rho_- t}\right). \tag{3}$$

The solution $Z(t)$ is dependent on $\rho_{\pm}$ that, in turn, depends on a number of system parameters, including thermal reversion rates and interaction rates, that can be difficult to elucidate experimentally. Thus, if we assume that two forms of P_A naturally exist (as has been postulated experimentally [7,8]), we can set $\delta = \gamma = 0$ in equation (2) such that $\rho_+ = -(k_1 + k_2 + \beta_1)$ and $\rho_- = -(k_1 + k_2 + \beta_2)$. This is important, since we can estimate the values of β_k from our thermal reversion optimisation algorithm allowing us to use our approach to analyse these systems.

All that is left, then, is to calculate the value of the constants A, B, C, and D. Importantly, we wish to know how the percentage of active photoreceptor changes with time. Thus, we can rescale (3) using $\hat{x} = x/P_0$ for Z and the exponential constants. Therefore, we get

$$\begin{aligned} \hat{Z}(0) &= \hat{A} + \hat{B} = \sum_{k=1}^{2} \alpha_k c_{\lambda_1}^k = c_{\lambda_1}, \\ \lim_{t\to\infty} \hat{Z}(t) &= \hat{C} + \hat{D} = \sum_{k=1}^{2} \alpha_k c_{\lambda_2}^k = c_{\lambda_2}, \\ \text{with } \sum_{k=1}^{2} \alpha_k &= 1, \end{aligned} \tag{4}$$

where $c_{\lambda_w} = c^A(\boldsymbol{\sigma}, \boldsymbol{\Phi}, \boldsymbol{\alpha}, \boldsymbol{\beta}, \Omega_P, \{(t \to \infty, z^{\lambda}_{\lambda_w})\})$ (as explained in the main text) and, similarly, $c^k_{\lambda_w} = c^A_k(\boldsymbol{\sigma}, \boldsymbol{\Phi}, \boldsymbol{\alpha}, \boldsymbol{\beta}, \Omega_P, \{(t \to \infty, z^{\lambda}_{\lambda_w})\})$ is the steady state fraction of P_A subpopulation k. From our thermal reversion optimisation routine, as well as obtaining the multiple thermal reversion rates, we also obtain the percentage of the active photoreceptor population undergoing that particular thermal reversion reaction, α_1 and $\alpha_2 = 1 - \alpha_1$.

Thus, we can satisfy (4) by

$$\begin{aligned} \hat{A} &= \alpha_1 c^1_{\lambda_1} \\ \hat{B} &= (1-\alpha_1) c^2_{\lambda_1} \\ \hat{C} &= \alpha_1 c^1_{\lambda_2} \\ \hat{D} &= (1-\alpha_1) c^2_{\lambda_2}. \end{aligned}$$

Finally, by setting $\alpha = \alpha_1$ and $k_i = \sum_\lambda \sigma^\lambda_i z^\lambda_{\lambda_w}$ for a given light treatment λ_w, we obtain

$$\begin{aligned} & c^A(\boldsymbol{\sigma}, \boldsymbol{\Phi}, \boldsymbol{\alpha}, \boldsymbol{\beta}, \{(t \to \infty, z^{\lambda}_{\lambda_1})\}, \{(t, z^{\lambda}_{\lambda_2})\}) = \\ & \alpha c^1_{\lambda_1} e^{\{-t[\sum_\lambda(\sigma^\lambda_A + \sigma^\lambda_I) z^\lambda_{\lambda_2} + \beta_1]\}} + (1-\alpha) c^2_{\lambda_1} e^{\{-t[\sum_\lambda(\sigma^\lambda_A + \sigma^\lambda_I) z^\lambda_{\lambda_2} + \beta_2]\}} \\ & + \alpha c^1_{\lambda_2}(1 - e^{\{-t[\sum_\lambda(\sigma^\lambda_A + \sigma^\lambda_I) z^\lambda_{\lambda_2} + \beta_1]\}}) + (1-\alpha) c^2_{\lambda_2}(1 - e^{\{-t[\sum_\lambda(\sigma^\lambda_A + \sigma^\lambda_I) z^\lambda_{\lambda_2} + \beta_2]\}}) \end{aligned} \tag{5}$$

or

$$\begin{aligned} & c^A(\boldsymbol{\sigma}, \boldsymbol{\Phi}, \boldsymbol{\alpha}, \boldsymbol{\beta}, \{(t \to \infty, z^{\lambda}_{\lambda_2})\}, \{(t, z^{\lambda}_{\lambda_1})\}) = \\ & \alpha c^1_{\lambda_2} e^{\{-t[\sum_\lambda(\sigma^\lambda_A + \sigma^\lambda_I) z^\lambda_{\lambda_1} + \beta_1]\}} + (1-\alpha) c^2_{\lambda_2} e^{\{-t[\sum_\lambda(\sigma^\lambda_A + \sigma^\lambda_I) z^\lambda_{\lambda_1} + \beta_2]\}} \\ & + \alpha c^1_{\lambda_1}(1 - e^{\{-t[\sum_\lambda(\sigma^\lambda_A + \sigma^\lambda_I) z^\lambda_{\lambda_1} + \beta_1]\}}) + (1-\alpha) c^2_{\lambda_1}(1 - e^{\{-t[\sum_\lambda(\sigma^\lambda_A + \sigma^\lambda_I) z^\lambda_{\lambda_1} + \beta_2]\}}). \end{aligned} \tag{6}$$

Thus, if $\alpha = 1$, then $B = D = 0$ and our system will only describe a single photoreceptor form with thermal reversion rate β_1. In Figures 3, 4 & 6 of the main text we show the results of our approach given different numbers of thermal reaction equations following (5) or (7).

1.2 Forms of steady-state percentages of active photoreceptor, c_{λ_w}

In this section we shall present some of the known forms for the steady state percentage of active photoreceptor in a population given illumination wavelength λ_w.

As noted in [2], in the absence of thermal reversion, synthesis and degradation, phytochrome dynamics are described by

$$\begin{aligned}\frac{dP_I}{dt} &= k_2 P_A - k_1 P_I,\\ \frac{dP_A}{dt} &= k_1 P_I - k_2 P_A.\end{aligned}$$

At steady-state, one finds that

$$c_{\lambda_w} = \frac{P_A}{P_A + P_I} = \frac{\sum_\lambda \sigma_I^\lambda z_{\lambda_w}^\lambda}{\sum_\lambda (\sigma_A^\lambda + \sigma_I^\lambda) z_{\lambda_w}^\lambda},$$

where $k_i = \sum_\lambda \sigma_i^\lambda z_{\lambda_w}^\lambda$ as before. When the photoreceptor undergoes a single thermal reversion reaction the system reads

$$\begin{aligned}\frac{dP_I}{dt} &= (k_2 + \beta) P_A - k_1 P_I,\\ \frac{dP_A}{dt} &= k_1 P_I - (k_2 + \beta) P_A,\end{aligned}$$

and

$$c_{\lambda_w} = \frac{\sum_\lambda \sigma_I^\lambda z_{\lambda_w}^\lambda}{\sum_\lambda (\sigma_A^\lambda + \sigma_I^\lambda) z_{\lambda_w}^\lambda + \beta},$$

where β is the thermal reversion rate.

In our study we discuss two further cases that will influence the distribution of active photoreceptors within a population, namely: the existence of two sub-populations of monomeric photoreceptor, and; the dimerisation of photoreceptors. Using the model above in equation (3), and setting $t = 0$ or $t \to \infty$, yields for the case of multiple photoreceptor sub-populations

$$\begin{aligned}c_{\lambda_w} &= \alpha c_{\lambda_w}^1 + (1 - \alpha) c_{\lambda_w}^2, \qquad (7)\\ c_{\lambda_w}^k &= \frac{\sum_\lambda \sigma_I^\lambda z_{\lambda_w}^\lambda}{\sum_\lambda (\sigma_A^\lambda + \sigma_I^\lambda) z_{\lambda_w}^\lambda + \beta_k},\end{aligned}$$

where β^k represents multiple thermal reversion rates. The form of $c_{\lambda_w}^k$ can be calculated from our model (equation (1)) at steady state when $\delta = \gamma = 0$.

To obtain the steady state percentage of active photoreceptor for the case of photoreceptor dimers, we use the model of [1] in the absence of synthesis and degradation reactions. The system reads

$$\begin{aligned}\frac{dP_{II}}{dt} &= (k_2 + \beta_1)P_{AI} - 2k_1 P_{II}, \\ \frac{dP_{AI}}{dt} &= 2(k_2 + \beta_2)P_{AA} + 2k_1 P_{II} - (k_1 + k_2 + \beta_1)P_{AI}, \\ \frac{dP_{AA}}{dt} &= k_1 P_{AI} - 2(k_2 + \beta_2)P_{AA},\end{aligned}$$

where P_{II} is the $P_I - P_I$ homodimer, P_{AI} is the $P_A - P_I$ heterodimer, and P_{AA} is the $P_A - P_A$ homodimer. From these equations we obtain

$$\begin{aligned}c_{\lambda_w} &= c^1_{\lambda_w} + 2c^2_{\lambda_w}, \qquad (8)\\ c^1_{\lambda_w} &= \frac{2\sum_\lambda(\sigma^\lambda_I z^\lambda_{\lambda_w})[\sum_\lambda(\sigma^\lambda_A z^\lambda_{\lambda_w}) + \beta_2]}{\sum_\lambda(\sigma^\lambda_I z^\lambda_{\lambda_w})^2 + [\sum_\lambda(\sigma^\lambda_A z^\lambda_{\lambda_w}) + \beta_2][2\sum_\lambda(\sigma^\lambda_I z^\lambda_{\lambda_w}) + \sum_\lambda(\sigma^\lambda_A z^\lambda_{\lambda_w}) + \beta_1]}, \\ c^2_{\lambda_w} &= \frac{\sum_\lambda(\sigma^\lambda_I z^\lambda_{\lambda_w})}{2[\sum_\lambda(\sigma^\lambda_A z^\lambda_{\lambda_w}) + \beta_2]} c^1_{\lambda_w},\end{aligned}$$

where $c^1_{\lambda_w}$ is the steady-state percentage of P_A-P_I heterodimers and $c^2_{\lambda_w}$ is the steady-state percentage of P_A-P_A homodimers. Here, the multiplication by $n_c = 2$ of $c^2_{\lambda_w}$ is required as the homodimer complex contains two light-absorbing chromophores within it's structure and $\sum_{k=1}^2 c^k(\boldsymbol{\sigma}, \boldsymbol{\Phi}, \boldsymbol{\alpha}, \boldsymbol{\beta}, \Omega_P, \Omega_E) = n_c$ from equation (1) of the main text.

Depending on the case under investigation or what is known about the photoreceptor from previous studies will determine which form of c_{λ_w} needs to be used within the algorithm. A further example can be found in Section 1.5.1.

1.3 Relating c's to X's in Verméglio method

In the main text, we discuss the construction of search spaces for a photoreceptor's photoconversion cross-sections due to unknown parameters, such as the percentage of (in)active photoreceptor, quantum yields and thermal reversion rates. However, if some of these are known (for example there is no thermal reversion and absorption spectra show that 100% of the photoreceptor is in a particular confirmation) then the values of X_1 and X_2 can be calculated directly. Here, in the case of a photoreceptor with two light regulated species, we show how the X's are related to the steady-state fraction of active photoreceptor after saturating light conditons (c_{λ_w}).

From the definition of absorption spectra and how the photoconversion cross-sections are related to multiple spectra, we have, for any number of available chromophores n_c

$$A_s(\lambda|\Omega_P, \{(t \to \infty, z^{\lambda}_{\lambda_A})\}) = 2.303 l c_{tot}[(n_c - c_{\lambda_A})\epsilon^{\lambda}_I + c_{\lambda_A}\epsilon^{\lambda}_A],$$
$$A_s(\lambda|\Omega_P, \{(t \to \infty, z^{\lambda}_{\lambda_I})\}) = 2.303 l c_{tot}[(n_c - c_{\lambda_I})\epsilon^{\lambda}_I + c_{\lambda_I}\epsilon^{\lambda}_I], \quad (9)$$

where

$$\begin{aligned} n_c\hat{\epsilon}^{\lambda}_I &= 2.303 l c_{tot} n_c \epsilon^{\lambda}_I \\ &= A_e(\lambda|\Omega_P, \{(t \to \infty, z^{\lambda}_{\lambda_A})\}) + X_1(A_e(\lambda|\Omega_P, \{(t \to \infty, z^{\lambda}_{\lambda_I})\}) \\ &\qquad - A_e(\lambda|\Omega_P, \{(t \to \infty, z^{\lambda}_{\lambda_A})\})), \\ n_c\hat{\epsilon}^{\lambda}_A &= 2.303 l c_{tot} n_c \epsilon^{\lambda}_A \\ &= A_e(\lambda|\Omega_P, \{(t \to \infty, z^{\lambda}_{\lambda_A})\}) + X_2(A_e(\lambda|\Omega_P, \{(t \to \infty, z^{\lambda}_{\lambda_I})\}) \\ &\qquad - A_e(\lambda|\Omega_P, \{(t \to \infty, z^{\lambda}_{\lambda_A})\})). \end{aligned} \quad (10)$$

By assuming that experimental spectra share the same relationship to their extinction coefficients as simulated spectra (i.e. that our model is correct) and substituting (9) into (10), one finds that

$$n_c\hat{\epsilon}^{\lambda}_I = \hat{\epsilon}^{\lambda}_I[n_c - c_{\lambda_A} + X_1(c_{\lambda_A} - c_{\lambda_I})] + \hat{\epsilon}^{\lambda}_A[c_{\lambda_A} - X_1(c_{\lambda_A} - c_{\lambda_I})],$$
$$n_c\hat{\epsilon}^{\lambda}_A = \hat{\epsilon}^{\lambda}_I[n_c - c_{\lambda_A} + X_2(c_{\lambda_A} - c_{\lambda_I})] + \hat{\epsilon}^{\lambda}_A[c_{\lambda_A} - X_2(c_{\lambda_A} - c_{\lambda_I})], \quad (11)$$

which leads to

$$c_{\lambda_A} - X_1(c_{\lambda_A} - c_{\lambda_I}) = 0,$$
$$c_{\lambda_A} - X_2(c_{\lambda_A} - c_{\lambda_I}) = n_c, \quad (12)$$

and so

$$X_1 = \frac{c_{\lambda_A}}{c_{\lambda_A} - c_{\lambda_I}},$$
$$X_2 = \frac{c_{\lambda_A} - n_c}{c_{\lambda_A} - c_{\lambda_I}}. \quad (13)$$

Furthermore, these values satisfy the constraints

$$n_c X_1 + c_{\lambda_A}(X_2 - X_1) = 0,$$
$$n_c X_1 + c_{\lambda_I}(X_2 - X_1) = n_c.$$

1.4 Application to a simpler photoreceptor system

As well as the phytochromes, further photoreceptors are studied in plant and optogenetic systems. For example, blue light- and UV-B-responsive photoreceptors have been used in a range of optogenetic tools (see the review in [9]). Importantly, compared to phytochromes, these systems only respond to one region of the light spectrum and can only be de-activated by periods of darkness and thermal relaxation. Consequently, the mathematics can be simplified. Here, we will provide the equations and how to find the optimal solution.

First, since these photoreceptors have only one light-induced state, the equation for absorption can be written as

$$A_s(\lambda|\{(t\to\infty,z^{\lambda}_{\lambda_{w_1}})\},\{(t,z^{\lambda}_{\lambda_{w_2}})\}) = 2.303 l c_{tot} c^I(\boldsymbol{\sigma},\boldsymbol{\Phi},\boldsymbol{\alpha},\boldsymbol{\beta},\{(t\to\infty,z^{\lambda}_{\lambda_{w_1}})\},\{(t,z^{\lambda}_{\lambda_{w_2}})\})\frac{\sigma^{\lambda}_I}{\Phi_I}, \quad (14)$$

where σ^{λ}_I and Φ_I are the photoconversion cross-sections and quantum yield of the inactive photoreceptor to reach the active confirmation. Note that here $c^I = n_c - c^A$ with $n_c = 1$ as the photoreceptor exists as a monomer.

Since, after prolonged darkness, the percentage of active photoreceptor is zero and there is no light reaction-induced absorption, we can only obtain the cross section after illumination of activating light conditions. Thus, $c_{\lambda_I} = 0$ and

$$c^A(\boldsymbol{\sigma},\boldsymbol{\Phi},\boldsymbol{\alpha},\boldsymbol{\beta},\{(t\to\infty,z^{\lambda}_{\lambda_I})\},\{(t,z^{\lambda}_{\lambda_A})\}) = c^1_{\lambda_A}(1-\mathrm{e}^{-t(\sum_\lambda \sigma^{\lambda}_I z^{\lambda}_{\lambda_A}+\beta_1)}) \quad (15)$$

in the assumed case where a single population of active photoreceptor exist. Furthermore, terms describing the fraction of active photoreceptor after saturating light conditions ($t\to\infty$) are simplified as well. For example,

$$c_{\lambda_A} = \frac{\sum_\lambda \sigma^{\lambda}_I z^{\lambda}_{\lambda_A}}{\sum_\lambda \sigma^{\lambda}_I z^{\lambda}_{\lambda_A}+\beta}.$$

Finally, we note that the Verméglio method is simplified also, since

$$\hat{\epsilon}^{\lambda}_I = 2.303 l c_{tot}\epsilon^{\lambda}_I = A_e(\lambda|\Omega_P,\{(t\to\infty,z^{\lambda}_{\lambda_A})\})+ X(A_e(\lambda|\{(t\to\infty,z^{\lambda}_{\lambda_I})\},\{(0,z^{\lambda}_{\lambda_A})\}) - A_e(\lambda|\Omega_P,\{(t\to\infty,z^{\lambda}_{\lambda_A})\})), \quad (16)$$

which upon substitution into (14) with $A_e(\lambda|\{(t\to\infty,z^{\lambda}_{\lambda_I})\},\{(0,z^{\lambda}_{\lambda_A})\}) = 0$ results in

$$A_s(\lambda|\Omega_P,\{(t\to\infty,z^\lambda_{\lambda_A})\}) = (1-c_{\lambda_A})(1-X)A_e(\lambda|\Omega_P,\{(t\to\infty,z^\lambda_{\lambda_A})\}),$$

and

$$\begin{aligned}&(1-c_{\lambda_A})(1-X)=1,\\ &X=\frac{c_{\lambda_A}}{c_{\lambda_A}-1}.\end{aligned}$$

As with the case of phytochromes, a search space for $\hat{\epsilon}^\lambda_I$ can be obtained by varying Φ_I and calculating all possible values of X_2. Using this search space, the optimal Φ_I and set of σ^λ_I can be obtained by minimizing the differences between simulated data and measured absorption spectra.

1.5 Generalising method to photocycles containing N species

Whilst in the previous section we have simplified the analysis of the main paper for a photoreceptor with a single state, we can also generalise our method to N-step photocycles. Such complex photoreactions have already been shown to govern the dynamics of phytochromes [10,11]. Here, we shall first present the conditions that need to be satisfied to determine the search spaces for $\hat{\epsilon}^\lambda_i$ and values for $R^{\max}_i$ (the upper bound for the quantum yield ratios) for $i=1,...,N$ before illustrating these results for a 3-step cycle that incorporates the phytochrome species Lumi-R [10–12]. Note that here we assume there is no dimerisation between photoreceptors such that $n_c=\sum_{k=1}^N c^k(\boldsymbol{\sigma},\boldsymbol{\Phi},\boldsymbol{\alpha},\boldsymbol{\beta},\Omega_P,\Omega_E)=1$.

From equation (1) of the main text, we see that absorption spectra can be described as a linear combination of extinction coefficients such that

$$A_s(\lambda|\Omega_P,\Omega_E)=\sum_{k=1}^N c^k(\boldsymbol{\sigma},\boldsymbol{\Phi},\boldsymbol{\alpha},\boldsymbol{\beta},\Omega_P,\Omega_E)\hat{\epsilon}^\lambda_k=\mathbf{c}_t\cdot\hat{\boldsymbol{\epsilon}}^\lambda, \tag{17}$$

where $\hat{\epsilon}^\lambda_k=2.303lc_{tot}\epsilon^\lambda_k$. Furthermore, by again assuming that our model of absorption is correct such that measured spectra share the same relationship with extinction coefficients as simulated spectra, we can rearrange equation (4) of the main text to read

$$\begin{aligned}\hat{\epsilon}^\lambda_I&=(1-X_1)A_e(\lambda|\Omega_P,\{(t\to\infty,z^\lambda_{\lambda_A})\})+X_1A_e(\lambda|\Omega_P,\{(t\to\infty,z^\lambda_{\lambda_I})\}),\\ \hat{\epsilon}^\lambda_A&=(1-X_2)A_e(\lambda|\Omega_P,\{(t\to\infty,z^\lambda_{\lambda_A})\})+X_2A_e(\lambda|\Omega_P,\{(t\to\infty,z^\lambda_{\lambda_I})\}),\\ \hat{\boldsymbol{\epsilon}}^\lambda&=\begin{pmatrix}\hat{\epsilon}^\lambda_I\\ \hat{\epsilon}^\lambda_A\end{pmatrix}=\begin{pmatrix}1-X_1 & X_1\\ 1-X_2 & X_2\end{pmatrix}\begin{pmatrix}A_e(\lambda|\Omega_P,\{(t\to\infty,z^\lambda_{\lambda_A})\})\\ A_e(\lambda|\Omega_P,\{(t\to\infty,z^\lambda_{\lambda_I})\})\end{pmatrix}=\boldsymbol{\kappa}\mathbf{A}.\end{aligned} \tag{18}$$

Thus, from equations (17) and (18) we obtain an expression for any steady-state absorption spectra after illumination with light condition $z^{\lambda}_{\lambda_k}$

$$A_s(\lambda|\Omega_P, \{(t \to \infty, z^{\lambda}_{\lambda_k})\}) = \mathbf{c}_\infty \cdot (\boldsymbol{\kappa}\mathbf{A})_k, \tag{19}$$

where $(\boldsymbol{\kappa}\mathbf{A})_k$ is the k-th row of $\boldsymbol{\kappa}\mathbf{A}$. Solving this system for $z^{\lambda}_{\lambda_A}$ and $z^{\lambda}_{\lambda_I}$, and using the relation that $c^A_\infty + c^I_\infty = 1$, provides us with the functions of equation (6) in the main text to obtain values of X_1, X_2 and $R^{\max}$.

Given this reformulation of the problem, one can now generalize the system to a reaction cycle containing N species. In this instance we write

$$\begin{aligned}
\mathbf{c}_\infty &= (c^1_\infty, c^2_\infty, ..., c^N_\infty),\\
\mathbf{A} &= \begin{pmatrix} A_e(\lambda|\Omega_P, \{(t \to \infty, z^{\lambda}_{\lambda_N})\}) \\ A_e(\lambda|\Omega_P, \{(t \to \infty, z^{\lambda}_{\lambda_{N-1}})\}) \\ \vdots \\ A_e(\lambda|\Omega_P, \{(t \to \infty, z^{\lambda}_{\lambda_1})\}) \end{pmatrix},\\
\boldsymbol{\kappa} &= \begin{pmatrix} 1-X_1 & \gamma^1_1 X_1 & \gamma^2_1 X_1 & \cdots & (1-\sum_{i=1}^{N-2}\gamma^i_1)X_1 \\ 1-X_2 & \gamma^1_2 X_2 & \gamma^2_2 X_1 & \cdots & (1-\sum_{i=1}^{N-2}\gamma^i_2)X_2 \\ \vdots & \vdots & \vdots & \ddots & \vdots \\ 1-X_N & \gamma^1_N X_N & \gamma^2_N X_1 & \cdots & (1-\sum_{i=1}^{N-2}\gamma^i_N)X_N \end{pmatrix},
\end{aligned} \tag{20}$$

where z_{λ_j} are the N light conditions required to obtain the N steady state absorption spectra for each species and γ^j_i are scaling factors such that $\sum_j \kappa_{kj} = 1$.

From equation (20) we thus have, for any steady-state absorption spectra,

$$\begin{aligned}
A_e(\lambda|\Omega_P, \{(t \to \infty, z^{\lambda}_{\lambda_i})\}) &= \sum_{j=1}^{N} c^j_\infty \left[(1-X_j)\mathbf{A}_1 + \sum_{i=1}^{N-2}\gamma^j_i X_j \mathbf{A}_{i+1} + \mathbf{A}_N X_j\left(1-\sum_{i=1}^{N-2}\gamma^i_j\right)\right],\\
&= \mathbf{A}_1 \sum_{j=1}^{N} c^j_\infty(1-X_j) + \sum_{j=1}^{N} c^j_\infty X_j \sum_{i=1}^{N-2}\gamma^j_i \mathbf{A}_{i+1} + \mathbf{A}_N \sum_{j=1}^{N} c^j_\infty X_j\left(1-\sum_{i=1}^{N-2}\gamma^i_j\right),\\
&= A_e(\lambda|\Omega_P, \{(t \to \infty, z^{\lambda}_{\lambda_N})\}) + \dots\\
&\quad + (A_e(\lambda|\Omega_P, \{(t \to \infty, z^{\lambda}_{\lambda_1})\}) - A_e(\lambda|\Omega_P, \{(t \to \infty, z^{\lambda}_{\lambda_N})\}))\sum_{j=1}^{N} c^j_\infty X_j\\
&\quad + \sum_{i=1}^{N-2}(A_e(\lambda|\Omega_P, \{(t \to \infty, z^{\lambda}_{\lambda_{N-i}})\}) - A_e(\lambda|\Omega_P, \{(t \to \infty, z^{\lambda}_{\lambda_1})\}))\sum_{j=1}^{N} c^j_\infty \gamma^i_j X_j.
\end{aligned} \tag{21}$$

From this, we now obtain sets of simultaneous equations that satisfy this relationship and can be solved to provide us with values of X_i and $R_i^{\max} = \Phi_i/\Phi_1$, the ratio of quantum yields for each species relative to the inactive state. For example, if $A_e(\lambda|\Omega_P, \{(t \to \infty, z^{\lambda}_{\lambda_i})\}) = A_e(\lambda|\Omega_P, \{(t \to \infty, z^{\lambda}_{\lambda_N})\})$ then

$$\begin{aligned} &\sum_{j=1}^{N} c^j_\infty X_j = 0, \\ &\sum_{j=1}^{N} c^j_\infty X_j \gamma^i_j = 0, \text{ for i} \in \text{[2,N-1]}. \end{aligned} \tag{22}$$

In the case of two species, and using $c^1_\infty + c^2_\infty = 1$ we obtain equivalent relations to those of equation (6) in the main text.

1.5.1 Example: the three-species cycle of phytochromes

As an illustration of the N species extension outlined above we can now obtain the relationships for a three species phytochrome system. In this system, inactive P_I is photoconverted into the Lumi-R (L_I) state under red light that goes on to form P_A. As with previously, P_A can then revert back to P_I using far-red light or thermally in darkness [10–12]. The ODE model of this system is then

$$\begin{aligned} \frac{dP_I}{dt} &= (k_2 + \beta_1)P_A + k_3 L_I - k_1 P_I, \\ \frac{dL_I}{dt} &= k_1 P_I - (k_3 + \beta_2) L_I, \\ \frac{dP_A}{dt} &= \beta_2 L_I - (k_2 + \beta_1) P_A, \end{aligned} \tag{23}$$

where k_i are light regulated reactions and β_j are thermal reactions.

Consequently, one can obtain analytical expression for the steady states of the three components

$$\begin{aligned} \hat{P}_I &= \frac{(k_2 + \beta_1)(k_3 + \beta_2)}{(k_1 + k_3 + \beta_2)(k_2 + \beta_1) + k_1\beta_2}, \\ \hat{L}_I &= \frac{k_1(k_2 + \beta_1)}{(k_1 + k_3 + \beta_2)(k_2 + \beta_1) + k_1\beta_2}, \\ \hat{P}_A &= \frac{k_1\beta_2}{(k_1 + k_3 + \beta_2)(k_2 + \beta_1) + k_1\beta_2}. \end{aligned} \tag{24}$$

Then, given that $k_i = \sum_\lambda \sigma_i^\lambda z_{\lambda_w}^\lambda = \Phi_i \sum_\lambda \epsilon_i^\lambda z_{\lambda_w}^\lambda = \Phi_I S_i^w$ as in equation (8) of the main text, where σ_i^λ are the photoconversion cross-sections, Φ_i are the quantum yields and ϵ_i^λ are the extinction coefficients of each species $i \in \{I, L, A\}$, we can rewrite these expressions as

$$
\begin{aligned}
\hat{P}_I &= c_\infty^I(\boldsymbol{\sigma}, \boldsymbol{\Phi}, \boldsymbol{\beta}, z_{\lambda_w}) = c_{\lambda_w}^I \\
&= \frac{(\Phi_A S_A^w + \beta_1)(\Phi_L S_L^w + \beta_2)}{(\Phi_I S_F^w + \Phi_L S_L^w + \beta_2)(\Phi_A S_A^w + \beta_1) + \Phi_I S_I^w \beta_2}, \\
\hat{L}_I &= c_\infty^L(\boldsymbol{\sigma}, \boldsymbol{\Phi}, \boldsymbol{\beta}, z_{\lambda_w}) = c_{\lambda_w}^L \\
&= \frac{\Phi_I S_I^w (\Phi_A S_A^w + \beta_1)}{(\Phi_I S_I^w + \Phi_L S_L^w + \beta_2)(\Phi_A S_A^w + \beta_1) + \Phi_I S_I^w \beta_2}, \\
\hat{P}_A &= c_\infty^A(\boldsymbol{\sigma}, \boldsymbol{\Phi}, \boldsymbol{\beta}, z_{\lambda_w}) = c_{\lambda_w}^A \\
&= \frac{\Phi_I S_I^w \beta_2}{(\Phi_I S_I^w + \Phi_L S_L^w + \beta_2)(\Phi_A S_A^w + \beta_1) + \Phi_I S_I^w \beta_2}.
\end{aligned}
\tag{25}
$$

Using the conditions in equation (22) we then have

$$
\begin{aligned}
& c_{\lambda_A}^I X_1 + c_{\lambda_A}^L X_2 + c_{\lambda_A}^A X_3 = 0, \\
& c_{\lambda_A}^I \gamma_1^1 X_1 + c_{\lambda_A}^L \gamma_2^1 X_2 + c_{\lambda_A}^A \gamma_3^1 X_3 = 0,
\end{aligned}
\tag{26}
$$

which, upon substitution of equation (25) gives

$$
\begin{aligned}
& \left(\Phi_A S_A^A + \beta_1\right)\left(\Phi_L S_L^A + \beta_2\right) X_1 + \Phi_I S_I^A \left(\Phi_A S_A^A + \beta_1\right) X_2 \\
& \qquad + \beta_2 \Phi_A S_A^A X_3 = 0, \\
& \left(\Phi_A S_A^A + \beta_1\right)\left(\Phi_L S_L^A + \beta_2\right) \gamma_1^1 X_1 + \Phi_I S_A^I \left(\Phi_A S_A^A + \beta_1\right) \gamma_2^1 X_2 \\
& \qquad + \beta_2 \Phi_A S_A^A \gamma_3^1 X_3 = 0.
\end{aligned}
\tag{27}
$$

Then substituting the quantum yield ratios $R_A = \Phi_A / \Phi_I$ and $R_L = \Phi_L / \Phi_I$ and solving the equations one obtains

$$
\begin{aligned}
R_A &= \frac{\gamma_2^1 \beta_1 X_2 + \gamma_3^1 \beta_2 X_3 - \gamma_1^1 (\beta_1 X_2 + \beta_2 X_3)}{\Phi_A S_A^A X_2 (\gamma_1^1 - \gamma_2^1)}, \\
R_L &= \frac{\gamma_3^1 (\beta_2 X_1 + \Phi_A S_A^A X_2) - \gamma_1^1 \beta_2 X_1 - \gamma_2^1 \Phi_A S_A^A X_2}{\Phi_A S_L^A X_1 (\gamma_1^1 - \gamma_3^1)}.
\end{aligned}
\tag{28}
$$

These simultaneous equations can now be solved numerically within the framework of our optimisation procedure to find the optimal quantum yields and photoconversion cross-sections for multiple phytochrome species. However, one should note that to analytically obtain these expressions becomes increasingly challenging as the number of species in the reaction cycle increases [13]. Thus, one may wish to consider other methods of numerically obtaining the percentages of subspecies (possibly through numerical integration of the ODEs given specific parameter values) followed by obtaining numerical solutions of the simultaneous constraint equations (equation (22)).

1.6 Calculation of phyB-N photoconversion cross-sections using Butler's method

In order to show the differences between our method and those previously used to estimate the photoconversion cross-sections of full-length phytochrome, we have calculated the quantum yields and photoconversion cross-sections using Butler's method. Essentially, using this method we are assuming that $c_{\lambda_I} = 0$ (there is no P_A after saturating exposure to λ_I), which means that $X_1 = 1$ from equation (11). The full derivation of the method can be found in [14] but we shall summarise it here.

The first step is to plot L against $ln\left[100E_t^{\lambda_w}\right]$, where $E_t^{\lambda_w}$ is described in equation (2) of the main text given illumination of light with wavelength λ_w during the experiment. Here,

$$L = \sum_{\lambda} \left| \frac{z^{\lambda} l_0 \Delta t (1 - \mathrm{e}^{-\bar{A}_{\lambda} l / l_0})}{\bar{A}_{\lambda} V_s} \right|$$

where z^{λ} is our photon distribution, l_0 is the cuvette width, l is the corrected pathlength, Δt is the time of illumination, V_s is the volume of the sample and $\bar{A}_{\lambda}$ is the intensity normalised absorption of a blank sample (see Supplementary Table 2 for these parameter values) [14, 15].

Upon calculating these functions, the aim is to determine for which value of L allows $100E_t^{\lambda_w} = 36.8$ (referred to as $L_{36.8}$, for an example see the result for our data in Supplementary Figure 6C) [14, 16]. The term $L_{36.8}$ refers to the amount of exposure required for absorption to decay to e^{-1} (~ 0.368) of the initial value under the assumption that illumination at λ_w leads to a single photoproduct (i.e. P_A) [16]. By taking $\frac{1}{L_{36.8}}$, we get the values $\sigma_I^{657} = 43.9$ m^2/mol and $\sigma_A^{735} = 10.5$ m^2/mol using our data calculated at the peaks of photon absorption (656 nm LEDs peak at 657 nm, 740 nm LEDs peak at 735 nm, see Supplementary Figure 2). These are in broad agreement with the scale of values for the photoconversion cross-sections at these wavelengths that we obtained from our algorithm (Supplementary Table 4), espe-

cially when compared with the 'Mancinelli' spectra where both our algorithm and Butler's method predict much smaller photoconversion cross-sections for phyB-N (see Figure 6 of the main text).

The next step is to perform the following derivations to obtain the quantum yields and a scaling factor between the absorption spectra and photoconversion cross-sections.

- $\frac{\Phi_I}{\Phi_A} = \frac{\sigma_I^{\lambda_A} A_e(\lambda_I|\Omega_P,\{(t\to\infty,z^{\lambda}_{\lambda_A})\})}{\sigma_A^{\lambda_I} A_e(\lambda_A|\Omega_P,\{(t\to\infty,z^{\lambda}_{\lambda_I})\})}$, where $A_e(\lambda_A|\Omega_P,\{(t \to \infty, z^{\lambda}_{\lambda_I})\})$ is the absorbance of our sample measured at wavelength λ_A after saturating inactivating light $z^{\lambda}_{\lambda_I}$ (and the same for activating conditions).

- $X_{eq}^{\lambda_A} = 1 - \left| \frac{A_e(\lambda_I|\{(t\to\infty,z^{\lambda}_{\lambda_I})\},\{(0,z^{\lambda}_{\lambda_A})\})}{A_e(\lambda_A|\Omega_P,\{(t\to\infty,z^{\lambda}_{\lambda_I})\})} \right| \left| 1 + \frac{\Phi_I}{\Phi_A} \right|^{-1}$. We obtain a value of $X_{eq}^{\lambda_A} = 0.7457$ that leads to $X_2 \sim -0.3410$.

- $\hat{\epsilon}^{\lambda}_A = \frac{A_e(\lambda|\Omega_P,\{(t\to\infty,z^{\lambda}_{\lambda_A})\}) - (1-X_{eq}^{\lambda_A}) A_e(\lambda|\Omega_P,\{(t\to\infty,z^{\lambda}_{\lambda_I})\})}{X_{eq}^{\lambda_A}}$ - below we shall relate this to (16) and show that they are equivalent.

- $\alpha_{\lambda_A} = \frac{A_e(\lambda_I|\Omega_P,\{(t\to\infty,z^{\lambda}_{\lambda_A})\})\alpha_{\lambda_I}}{A_e(\lambda_A|\Omega_P,\{(t\to\infty,z^{\lambda}_{\lambda_I})\}) X_{eq}^{\lambda_A}}$, where $\alpha_{\lambda_I} = \epsilon_I^{\lambda_A} = A_e(\lambda_A|\Omega_P,\{(t \to \infty, z^{\lambda}_{\lambda_I})\})$ is the photoconversion cross-section of the P_I photoreceptor sub-population.

- $\Phi_A = \sigma_A^{\lambda_I}/\alpha_{\lambda_A}$. By calculating α_{λ_A} one is able to obtain the quantum yield Φ_A and then from step 1 calculate Φ_I.

Following this procedure, we calculated that Φ_I = 0.006 and Φ_A = 0.008 (Supplementary Figure 6B), or roughly one fifth of the the values calculated by our optimisation algorithm (Figure 6 of the main text).

All that remains is to calculate the wavelength-dependent photoconversion cross-sections of σ_A^{λ} using step 2. This is related to the Verméglio method since

$$
\begin{aligned}
\hat{\epsilon}_A^\lambda &= \frac{A_e(\lambda|\Omega_P, \{(t \to \infty, z_{\lambda_A}^\lambda)\})}{X_{eq}^{\lambda_A}} - \frac{1 - X_{eq}^{\lambda_A}}{X_{eq}^{\lambda_A}} A_e(\lambda|\Omega_P, \{(t \to \infty, z_{\lambda_I}^\lambda)\}), \\
&= A_e(\lambda|\Omega_P, \{(t \to \infty, z_{\lambda_A}^\lambda)\}) - \frac{1 - X_{eq}^{\lambda_A}}{X_{eq}^{\lambda_A}} (A_e(\lambda|\Omega_P, \{(t \to \infty, z_{\lambda_I}^\lambda)\}) \\
&\quad - A_e(\lambda|\Omega_P, \{(t \to \infty, z_{\lambda_A}^\lambda)\})), \\
&\equiv A_e(\lambda|\Omega_P, \{(t \to \infty, z_{\lambda_A}^\lambda)\}) + X_2(A_e(\lambda|\Omega_P, \{(t \to \infty, z_{\lambda_I}^\lambda)\}) \\
&\quad - A_e(\lambda|\Omega_P, \{(t \to \infty, z_{\lambda_A}^\lambda)\})).
\end{aligned}
$$

The resulting spectra can be seen in Supplementary Figure 6A. What is obvious from this figure is that the wavelength-dependency of σ_A^λ using Butler's method is similar to that obtained from our algorithm (compare the solid and dashed lines of Supplementary Figure 6A), however the relative amplitudes and absolute values at the peak of the photoconversion cross-sections obtained from Butler's method are smaller than those obtained by our optimisation algorithm. Using the calculated set of photoconversion cross-sections and quantum yields, we simulated difference spectra and compared the output to our data and the simulated spectra obtained from our optimal values (Supplementary Figure 6D). From this figure it is clear to see that Butler's model has not calculated values that provide a close match to the time-dependent spectra obtained experimentally and that our optimal values predict.

1.7 Absorption spectra optimisation algorithm

Here, we provide pseudocode for our optimisation algorithm that aims to minimise the difference between simulated and measured absorption spectra for given sets of photoconversion cross-sections.

1. Define the upper and lower bound of the estimates. We have used:

   $\Phi_A \in \big(0.001, 1\big)$

   $R \in \big(0.001, R^{\max}\big)$

   (or Φ_i and R_i, $i \in [2, N]$ in the N species case)

2. Start optimization routine to minimize the difference between simulated and experimental absorption spectra. We used the 'Interior-point' algorithm of the *fmincon* optimization function in MATLAB.

3. Define the initial conditions. To do this we used the *rng* and *rand* functions within MATLAB to generate random values between the upper and lower bounds for each parameter.

a. set $\Phi_A = R\Phi_I$ (or $\Phi_i = R_i\Phi_1$ in the N species case).

b. calculate the values of X_1 and X_2 (or X_i) for given quantum yields (see note below).

c. interpolate search space of photoconversion cross-sections for these values of X's.

d. calculate the cost score

$$\Omega = \big((\mathbf{D}(t,\lambda) - \mathbf{S}(t,\lambda))/\nu_t^\lambda\big) \cdot \big((\mathbf{D}(t,\lambda) - \mathbf{S}(t,\lambda))/\nu_t^\lambda\big),$$

as defined by equation (9) of the main text.

e. If Ω is less than the previous estimate, keep it as the current best score and try the next parameter estimates.

f. Once Ω reaches pre-defined termination criteria, stop the optimization and return the minimal score. In our work we have set the termination criteria to be:

i. Maximum number of iterations ($'MaxIter'$) = 10000.

ii. Maximum number of function evaluations ($'MaxFunEvals'$) = 10000.

iii. Optimization tolerances ($'TolFun'$, $'TolCon'$, $'TolX'$) = 0.001.

4. Perform steps 3-4 the defined number of times. In our case 100 times.

5. Keep results with the best Ω.

6. If the algorithm is boot-strapped further, then re-start points 4-7.

From the resulting optimal parameter sets the mean σ^λ-spectra can be obtained by calculating X_1 and X_2 for the optimal Φ's and interpolating the search space of photoconversion cross-sections.

Note: As stated in the main text, the functions of X_1 and X_2 are implicit. This means that they need to solved numerically. In this work we have used the MATLAB numerical solver *vpasolve*. However, in some unforeseen cases, the numerical solver fails to converge to an accurate solution of X_1 and X_2 for a given set of $\mathbf{\Phi}$. At these points, we obtain numerical solutions in the local vicinity of the original $\mathbf{\Phi}$ values (perturbing the values by 0.0001 in each direction of the $\mathbf{\Phi}$-space) and then interpolate these values to obtain a solution of X_1 and X_2 at the original values of $\mathbf{\Phi}$. This, in effect, produces a continuous space of potential X_1 and X_2 values. Due to the small size of the perturbations, we believe that this process does not greatly impact our results.

1.8 Thermal reversion optimisation algorithm

The algorithm to calculate thermal reversion parameters works as follows:

1. Use equation (3) of the main text to set $A_t^D = \sum_{k=1}^{n} \alpha_k \mathrm{e}^{-\beta_k t_D}$. Define the number of n exponential functions that should be fit.

2. Define the upper and lower bound of our parameter estimates. We have used:

   $\alpha_k \in \big(0.0001, 1\big)$ such that $\sum_k \alpha_k = 1$.

   $\beta_k \in \big(0.0001, 0.01\big)$.

   For cases where a single thermal reversion rate is required, α can be fixed to 1 or β values can be fixed to zero.

3. Perform the optimisation routine 1000 times:

   a. define initial conditions for the parameter search as random points between the upper and lower bounds;

   b. use the 'Interior-point' option for $fmincon$ in MATLAB to minimise

$$\Omega = \big((\mathbf{E}_t^D - \mathbf{A}_t^D)/\zeta_t\big) \cdot \big((\mathbf{E}_t^D - \mathbf{A}_t^D)/\zeta_t\big)$$

   where E_t^D is described by equation (2) of the main text and ζ_t is the standard deviation of the data (similar to ν_t^λ above).

   c. once Ω reaches pre-defined termination criteria, stop the optimization and return the minimal score. In our work we have set the termination criteria to be:

      i. maximum number of iterations ($'MaxIter'$) = 10000.

      ii. maximum number of function evaluations ($'MaxFunEvals'$) = 10000.

      iii. optimization tolerances ($'TolFun'$, $'TolCon'$, $'TolX'$) = 0.001.

2 Experimental Methods

2.1 Protein purification

The phyB(1-651)-AviTag-His6 encoding plasmid pMH17 was engineered by PCR amplification of phytochrome B (phyB, Genbank accession No. NM_127435) from the plasmid pAL149 [17] using oligonucleotides oMH1 (5′-TTCCGAATTCATTAAAGAGGAGAAATTAACTATGGTTTCCGGAGTCGGGGGTAG-3′) and oMH38 (5′-TGACGCGGCCGCTTAATGGTGATGGTGATGATGTTCGTGCCATTCGATTTTCTGAGCTTCGAAGATGTCGTTCAGACCGCTACCAGAACCTGCACCTAACTCATCAATCCCCTG-3′) followed by subsequent cloning into plasmid p83 [18] using the

restriction enzymes NotI and EcoRI.

For expression of phyB(1-651)-AviTag-His6, the plasmid pMH17 and the plasmid p171 [18,19] coding for the enzymes Ho and PcyA for synthesis of phycocyanobilin were cotransformed into *E. coli* BL21 Star^{TM} (DE3) (Invitrogen, Carlsbad, CA, cat. no. C601003) and were selected in LB medium with 100 μgml^{-1} ampicillin and 40 μgml^{-1} kanamycin. The bacteria were grown at 30°C to an OD600 of 0.8 after which 50 μM biotin was added and expression was induced with 1 mM IPTG and 0.4% (w/v) arabinose. After expression at 18°C for 18h in darkness, the cells were harvested by centrifugation at 6,500 $\times$ g for 10 min, resuspended in lysis buffer (50 mM NaH_2PO_4, 300 mM NaCl, 10 mM imidazole, pH 8.0) and disrupted using a French Press (APV 2000, APV Manufacturing, Bydgoszcz, Poland) at 1,000 bar. The lysate was clarified by centrifugation at 30,000 $\times$ g for 30 min and loaded onto a Ni-NTA agarose column (Qiagen, Hilden, Germany, cat. no. 30210). After washing with 20 column volumes of wash buffer (50 mM NaH_2PO_4, 300 mM NaCl, 20 mM imidazole, pH 8.0), the purified protein was eluted in 3 column volumes of elution buffer (50 mM NaH_2PO_4, 300 mM NaCl, 250 mM imidazole, pH 8.0).

2.2 Protein analysis protocol

Final preparations of phyB-N containing the N-terminal 650 amino acids were stored in 50 mM sodium phosphate buffer containing 300 mM NaCl, pH 7.0. The concentration of phyB was determined using BCA assay and purity was analysed according to SDS-PAGE. Absorption measurements were performed using Agilent/Hewlett-Packard 8453 Diode-Array UV-Vis Spectrophotometer (Agilent Technologies, CA, USA).

2.3 Absorption spectra measurements of photoswitching

Photoswitching experiments were conducted by diluting phyB-N in a buffer (50 mM sodium phosphate buffer containing 300 mM NaCl, pH 7.0) to final concentration 0.34 mg/ml as determined from BCA analysis. This concentration is below an optical density of 0.2 in order to avoid inner filter effects and ensuring homogeneous illumination of the sample [20]. Absorption and illumination measurements were performed in 500 μl Hellma Quartz (Hellma GmbH & Co. KG, Müllheim, Germany) black absorption cuvettes that are 1cm thick. Illumination was performed either using 656 nm or 740 nm light emitting diodes (LEDs) at 20 cm distance yielding 11 μmol/m^{2}s flux rates, respectively. The photon distribution of the LEDs was determined using a Jobin Yvon-Spex Fluorolog 3.22 (Horiba Scientific, NJ, USA) in combination with micromax fibre optics. The head of the fibre was placed 1.5 m in front of the LED and spectra were recorded. Recording of red to far-red experiments were performed by placing the cuvet with phyB-N under 656 nm LEDs for 5

min followed by 740 nm illumination for different time periods. Absorption spectra were recorded after 10, 20, 30, 40, 50, 60, 80, 100, 120, 160, 200 s of 740 nm illumination. The far-red to red experiment was conducted similarly, starting with 8 min of 740 nm light followed by 5, 10, 15, 20, 25, 30, 40, 50, 60, 120 and 180 s periods of 656 nm illumination. Measurements were conducted at room temperature (∼22°C) and recorded in biological duplicate.

2.4 Thermal reversion experiments

After 5 min illumination of 656 nm light at 22°C, a cuvet containing phyB-N was placed in complete darkness within the spectrophotometer. Absorption spectra were obtained on a binary logarithmic scale starting at 42 seconds until a final measurement after 172,032 s (∼48 h). Measurements were conducted at 22°C in duplicate.

References

[1] C. Klose, F. Venezia, A. Hussong, S. Kircher, E. Schäfer, and C. Fleck. Systematic analysis of how phytochrome b dimerization determines its specificity. *Nature Plants*, 1:15090, 2015.

[2] A. L. Mancinelli. The physiology of phytochrome action. In *Photomorphogenesis in plants*, pages 211–269. R. E. Kendrick and G. M. H. Kronenberg eds., Dordrecht: Kluwer Academic Publishers, 1994.

[3] J. Rausenberger, A. Hussong, S. Kircher, D. Kirchenbauer, J. Timmer, F. Nagy, E. Schäfer, and C. Fleck. An integrative model for phytochrome B mediated photomorphogenesis: from protein dynamics to physiology. *PLoS ONE*, 5(5):e10721, 2010.

[4] J. Rausenberger, A. Tscheuschler, W. Nordmeier, F. Wüst, J. Timmer, E. Schäfer, C. Fleck, and A. Hiltbrunner. Photoconversion and nuclear trafficking cycles determine phytochrome A's response profile to far-red light. *Cell*, 146:813–825, 2011.

[5] W. Rüdiger, F. Thümmler, E. Cmiel, and S. Schneider. Chromophore structure of the physioloically active form (pfr) of phytochrome. *Proceedings of the National Academy of Sciences USA*, 80:6244–6248, 1983.

[6] L. Li and J. C. Lagarias. Phytochrome assembly. *Journal of Biological Chemistry*, 267:19204–19210, 1992.

[7] E. Sethe Burgie, T. Wang, A. N. Bussell, J. M. Walker, H. Li, and R. D. Vierstra. Crystallographic and electron microscopic analyses of a bacterial phytochrome

reveal local and global rearrangements during photoconversion. *Journal of Biological Chemistry*, 289:24573–24587, 2014.

[8] H. Takala, A. Björling, M. Linna, S. Westenhoff, and J. A. Ihalainen. Light-induced changes in the dimerization interface of bacteriophytochromes. *Journal of Biological Chemistry*, 290:16383–16392, 2015.

[9] H. M. Beyer, S. Naumann, W. Weber, and G. Radziwill. Optogenetic control of signaling in mammalian cells. *Biotechnology Journal*, 9, 2014.

[10] V. A. Sineshchekov. Photobiophysics and photobiochemistry of the heterogenous phytochrome system. *Biochimica et Biophysica Acta*, 1228:125–164, 1995.

[11] J. Dasgupta, R. R. Frontiera, K. C. Taylor, J. C. Lagarias, and R. A. Mathies. Ultrafast excited-state isomerization in phytochrome revealed by femtosecond stimulated raman spectroscopy. *Proceedings of the National Academy of Sciences USA*, 106:1784–1789, 2009.

[12] J. J. van Thor, K. L. Ronayne, and M. Towrie. Formation of the early photoproduct lumi-r of cyanobacterial phytochrome cph1 observed by ultrafast mid-infrared spectroscopy. *Journal of the American Chemical Society*, 129:126–132, 2007.

[13] A. Björling, O. Berntsson, H. Lehtivuori, H. Takala, A. J. Hughes, M. Panman, M. Hoernke, S. Niebling, L. Henry, R. Henning, I. Kosheleva, V. Chukharev, N. V. Tkachenko, A. Menzel, G. Newby, D. Khakhulin, M. Wulff, J. A. Ihalainen, and S. Westenhoff. Structural photoactivation of a full-length bacterial phytochrome. *Science Advances*, 2:e1600920, 2016.

[14] J. M. Kelly and J. C. Lagarias. Photochemistry of 124-kilodalton *avena* phytochrome under constant illumination *in vitro*. *Biochemistry*, 24:6003–6010, 1985.

[15] J. N. Demas. Luminescence quantum yields. an analysis of finite excitation bandpass errors. *Analytical Chemistry*, 45:992–994, 1973.

[16] H. E. Johns. Photochemical reactions in nucleic acids. *Methods in Enzymology*, 16:253–316, 1969.

[17] A. Levskaya, O. D. Weiner, W. A. Lim, and C. A. Voigt. Spatiotemporal control of cell signalling using a light-switchable protein interaction. *Nature*, 461(7266):997–1001, 2009.

[18] L. O. Essen, J. Mailliet, and J. Hughes. The structure of a complete phytochrome photosensory module in the pr ground state. *Proceedings of the National Academy of Sciences USA*, 105(38):14709–14714, 2008.

[19] F. T. Landgraf, C. Forreiter, A. H. Picó, T. Lamparter, and J. Hughes. Recombinant holophytochrome in *escherichia coli*. *FEBS Letters*, 508(3):459–462, 2001.

[20] G. Marriott and I. Parker, editors. *Biophotonics*. Number 360 in Methods in Enzymology. Academic Press, San Diego, CA., 2003.

Supplementary Tables

Supplementary Table 1: Parameter values used for test case simulations.

Parameter	Unit	Value
l	cm	1
c_{tot}	mol/l	0.2/70000
$k_{d1}^{R \to D}$	s^{-1}	0.005
$k_{d2}^{R \to D}$	s^{-1}	0.0005
$\lambda_A^{R \to FR}$	nm	665
$\lambda_A^{R \to G}$	nm	660
$\lambda_A^{R_1 \to R_2}$	nm	660
$\lambda_I^{FR \to R}$	nm	740
$\lambda_I^{G \to R}$	nm	550
$\lambda_I^{R_2 \to R_1}$	nm	680
$N_{\lambda_A}^{R \to FR}$	$\mu mol/m^2 s$	15
$N_{\lambda_A}^{R \to G}$	$\mu mol/m^2 s$	10
$N_{\lambda_A}^{R_1 \to R_2}$	$\mu mol/m^2 s$	10
$N_{\lambda_I}^{FR \to R}$	$\mu mol/m^2 s$	10
$N_{\lambda_I}^{G \to R}$	$\mu mol/m^2 s$	5
$N_{\lambda_I}^{R_2 \to R_1}$	$\mu mol/m^2 s$	10
t_{λ_I}	s	0:0.15:0.75
t_{λ_A}	s	0:0.05:0.25

c_{tot} = molecular concentration (mg/ml) / molecular weight (Da)
$\lambda_x^{L_1 \to L_2}$ = wavelength of light used for experiment from state L_1 to L_2
t_{λ_x} = time period for illumination of wavelength λ_X in s
N_λ is the light intensity in $\mu mol/m^2 s$

Supplementary Table 2: Parameters required for calculation of L in Butler's method using simulated data.

Parameter	Unit	Value
l	cm	1.06
l_0	cm	1
$\bar{A}_\lambda$	-	0.001
$V_{R \to G}$	mL	0.74
$V_{R \to FR}$	mL	0.78
$V_{R_1 \to R_2}$	mL	0.76
t_{λ_I}	s	[0:0.15:0.75, 1, 2, 5]
t_{λ_A}	s	[0:0.05:0.25, 0.5, 1, 2]

V is the estimated sample volume for each simulated experiment chosen such that output of Butler's model matches input parameters
t_{λ_x} = time period for illumination of wavelength λ_X

Supplementary Table 3: Parameters required for calculation of L in Butler's method from real data.

Parameter	Unit	Value
l	cm	1.06
l_0	cm	1
$\bar{A}_{\lambda_A}$	-	0.00017
$\bar{A}_{\lambda_I}$	-	0.00014
V_s	mL	0.3

Values of $\bar{A}_\lambda$ calculated according to [15].

Supplementary Table 4: Optimal photoconversion cross-sections for phyB-N.

Wavelength (nm)	σ_I (m^2/mol)	σ_A (m^2/mol)
500	4.953	0.468
510	5.798	0.352
520	6.957	0.468
530	9.076	0.697
540	11.99	1.035
550	15.79	1.599
560	20.80	2.335
570	28.47	3.458
580	39.81	5.138
590	53.20	7.043
600	63.90	9.135
610	72.42	11.63
620	86.92	14.94
630	113.8	20.60
640	145.2	26.73
650	165.2	32.01
660	146.0	34.86
670	86.21	36.19
680	32.66	39.37
690	8.840	44.99
700	1.627	50.98
710	*-0.52*	54.19
720	*-1.13*	50.36
730	*-0.97*	37.86
740	*-0.57*	22.18
750	*-0.20*	10.05
760	*-0.03*	3.243
770	0	0

Values in italics are negative and, hence, biologically infeasible. These can be set to 0 without altering simulations of absorption spectra due to the relatively much larger σ_A values.

Supplementary Figures

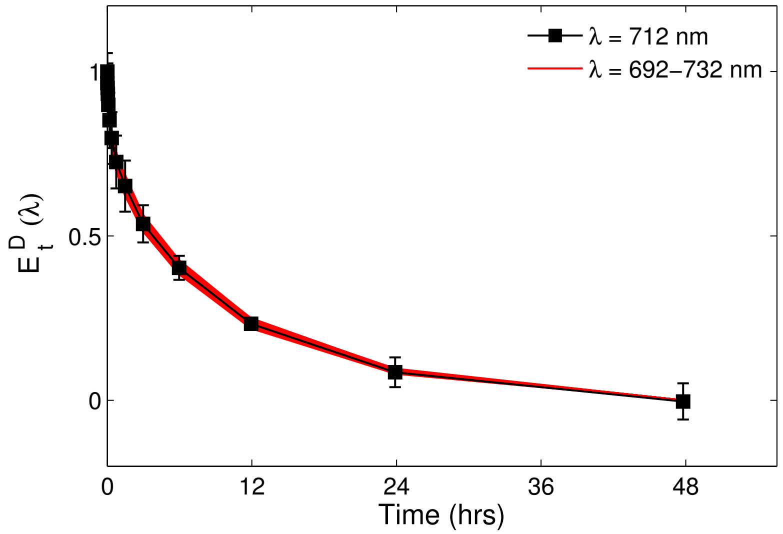

Supplementary Figure 1: Thermal reversion of phyB-N is independent of recorded P_A wavelength. Traces of thermal reversion as described by equation (2) of the main text over a 40 nm window around the peak wavelength of P_A.

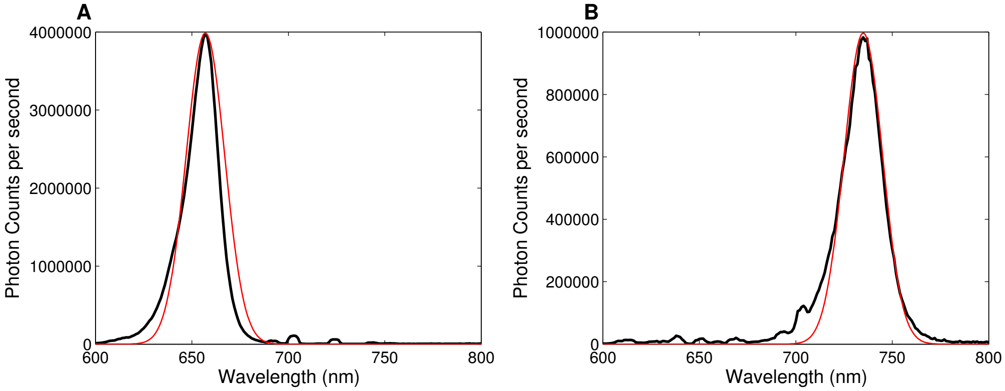

Supplementary Figure 2: Distribution of photons emitted by our LED light sources. Photon count measurements of the photons emitted by our (A) 656 nm and (B) 740 nm LED light sources (black lines). These are approximately the same as a Gaussian distribution with mean (A) 656 nm, (B) 740 nm and variance of 10 nm.

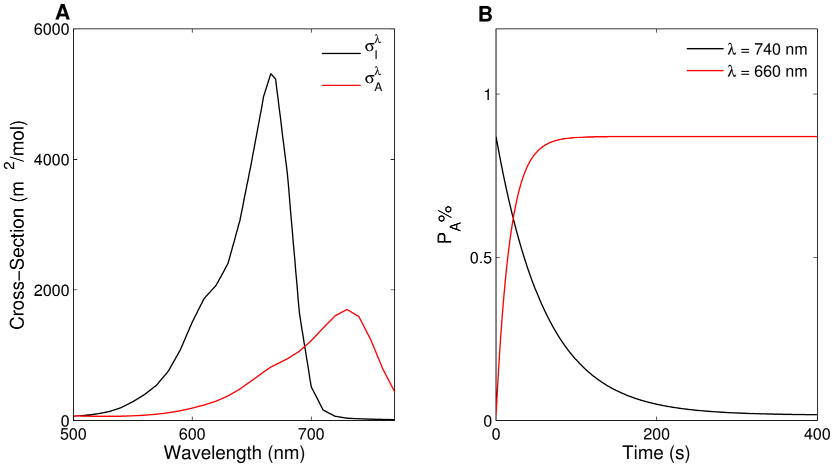

Supplementary Figure 3: Dynamics of full-length phytochromes as predicted by the 'Mancinelli' spectra. (A) photoconversion cross-sections for full-length phytochromes taken from [2]. Black line = σ_I; red line = σ_A. (B) The change of P_A percentage as obtained from equations (5) and (7) of Additional File 1 after red (black line) and far-red (red line) light conditions.

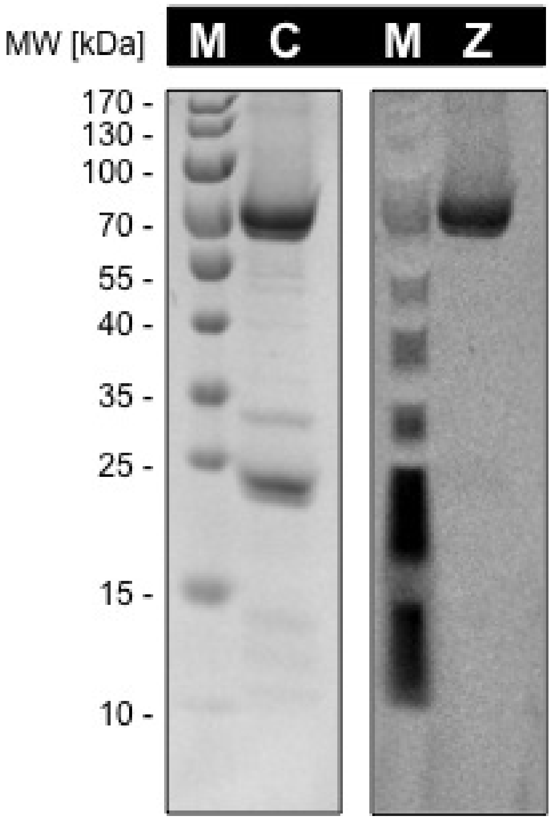

Supplementary Figure 4: Confirmation of phyB presence in our samples. Protein eluted from Ni-NTA purification was seperated by SDS-PAGE (10%), washed with dH_2O for 5 min and subjected to staining with 1 mM zinc acetate for 15 min to visualize the protein bound linear tetrapyrrole (right) followed by coomassie staining for total protein visualization (left). Zinc stain images were aquired by fluorescence upon UV light excitation. M = marker; C = coomassie stain; Z = zinc stain.

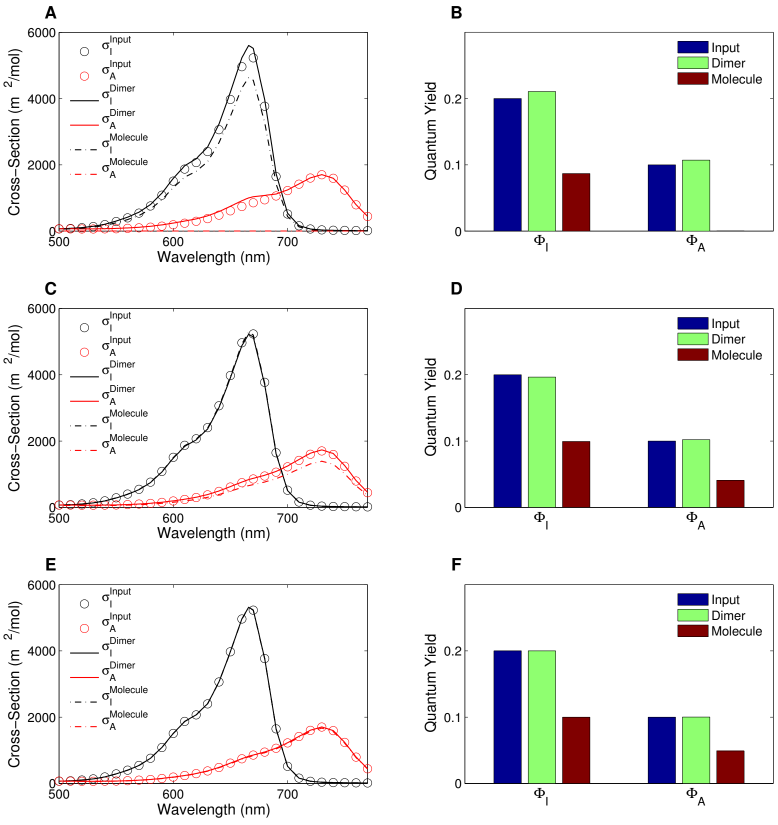

Supplementary Figure 5: Output of optimisation algorithm for a red/far-red photoreceptor undergoing two thermal reversion steps through complex formation. (A, C, E) Optimal photoconversion cross-sections using the correct form of c_t^k (equation (8); solid lines denoted 'Dimer') and incorrect form (equation (7); dashed lines denoted 'Molecule') compared to the input values (circles). Black lines/circles = σ_I; red lines/circles = σ_A. (B, D, F) Comparison of Φ_I and Φ_A obtained using optimisation algorithm for correct form ('Dimer') and incorrect ('Molecule') form of c_t^k to 'Input' values. (A, B) $[\alpha, \beta_1, \beta_2] = [0.7, 1, 0.1]$; (C, D) $[\alpha, \beta_1, \beta_2] = [0.7, 0.1, 0.01]$; (E, F) $[\alpha, \beta_1, \beta_2] = [0.7, 0.01, 0.001]$.

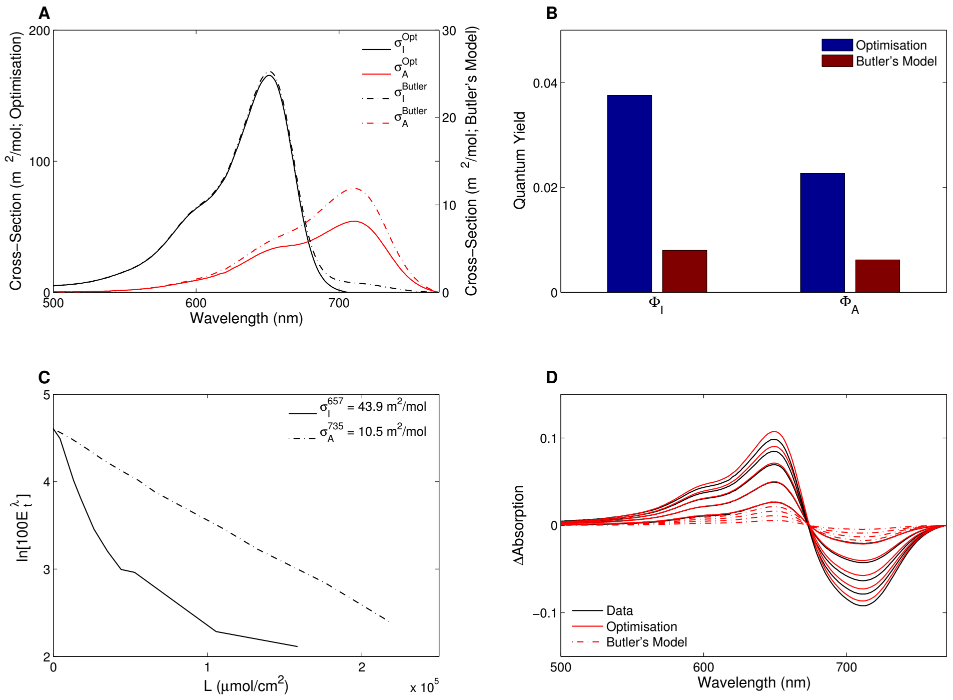

Supplementary Figure 6: photoconversion cross-sections for phyB-N obtained using the previously published Butler's model. (A) Comparison of photoconversion cross-sections obtained using Butler's model (dashed lines) compared to what our algorithm predicts for phyB-N (solid lines). (B) Values for the quantum yields of phyB-N obtained from our algorithm and derived from Butler's model. (C) Decay of absorption as a function of fluence. The value of $1/L_{36.8}$ provides the values of σ_k^λ. (D) Simulated spectra using Butler's model (dashed red line) compared to simulations using optimal values obtained from our algorithm (solid red line) and the obtained phyB-N data (black line).
